# Assessing community vulnerability to reduced vaccine impact in Uganda and Kenya: A spatial data analysis

**DOI:** 10.3310/nihropenres.13898.1

**Published:** 2025-03-17

**Authors:** Robinah Nalwanga, Agnes Natukunda, Ludoviko Zirimenya, Primus Chi, Henry Luzze, Alison M Elliott, Pontiano Kaleebu, Caroline L. Trotter, Emily L Webb

**Affiliations:** 1 Immunomodulation and Vaccines Focus Area, Vaccine Research Theme, Medical Research Council/Uganda Virus Research Institute and London School of Hygiene & Tropical Medicine Uganda Research Unit, Entebbe, Uganda; 2 Department of Infectious Disease Epidemiology and International Health, London School of Hygiene & Tropical Medicine, Keppel Street, London, WC1E 7HT, UK; 3 Department of Clinical Research, London School of Hygiene & Tropical Medicine, Keppel Street, London, WC1E 7HT, UK; 4 Centre for Geographic Medicine Research (Coast), Kenya Medical Research Institute-Wellcome Trust Research Programme, Kilifi, Kenya; 5 National Expanded Program on Immunization, Ministry of Health and Social Welfare, Kampala, Uganda; 6 Department of Veterinary Medicine, University of Cambridge, Cambridge, UK

**Keywords:** Vaccine impact, Vulnerability index, Vaccine coverage, Vulnerable communities, Uganda, Kenya

## Abstract

**Background:**

Despite global efforts to improve on vaccine impact, many African countries have failed to achieve equitable vaccine benefits. Reduced vaccine impact may arise from interplay between structural, social, and biological factors, that hinder communities from achieving full benefits from vaccination programs. However, the combined influence of these factors to reduced vaccine impact and the spatial distribution of vulnerable communities remains poorly understood. In this work, we developed a Community Vaccine Impact Vulnerability Index (CVIVI) that integrates data on multiple risk factors associated with impaired vaccine impact. The index identifies communities are at risk of reduced vaccine impact, and key factors contributing to their vulnerability.

**Methods:**

Vulnerability indicators were identified through literature review and grouped into structural, social, and biological domains. Using secondary data from Uganda and Kenya, we used percentile rank methodology to construct domain-specific and overall vulnerability indices. Correlation analysis was conducted to explore the relationship between indicators. Geo-spatial techniques were used to classify districts/counties from least to most vulnerable and to generate vulnerability maps.

**Results:**

Our findings revealed distinct geographical distribution of community vulnerability to reduced vaccine impact. In Kenya, the most vulnerable counties were clustered in the northeast and east, including Turkana, Mandera, and West Polot. In Uganda, vulnerability was more scattered, with the most vulnerable districts concentrated in the northeast (such as Amudat, Lamo) and southwest (such as Buliisa and Kyenjojo). Key factors contributing to high vulnerability in these counties/ districts cut across different domains, including long distance to the health facilities, low maternal education, low wealth quintile, high prevalence of malnutrition, limited access to postnatal care services, and limited access to mass media.

**Conclusions:**

The index is a potential tool for identifying vulnerable communities, and underlying causes of vulnerability, which guides the design of tailored strategies to improve vaccine impact among vulnerable communities.

## Plain Language Summary

Vaccination saves millions of lives every year; however, in many African countries, people are still dying from vaccine-preventable diseases. This is often due to low vaccine coverage and differences in how well individuals respond to vaccines. Several factors may contribute to these challenges, including poor access to healthcare services, high levels of poverty, and malnutrition, which collectively are likely to reduce benefits from vaccination programs. Identifying which communities at a risk of reduced vaccine impact and the main driver for their vulnerability requires data-driven approaches that integrate data on multiple risk factors into a single value. In this study, we developed the Community Vaccine Impact Vulnerability Index (CVIVI), which helps to identify geographical areas where communities are less likely to benefit fully from vaccines and vaccination programs. Using the CVIVI, we found out that factors such as high levels of poverty, low maternal education, limited access to mass media, and malnutrition often intersect in specific districts and counties, making these areas susceptible to reduced vaccine impact. For instance, counties such as Turkana in the northwest and Tana River in southeastern Kenya, along with Buliisa district in western Uganda and Amudat district in northern Uganda, were identified as most vulnerable. The index thus enables policymakers and researchers to identify communities at a risk of reduced vaccine impact and highlight barriers contributing to this vulnerability. With this information, the index would serve as a starting point for policymakers, implementers, researchers, and other stakeholders to understand vulnerability to vaccine impact, and design better tailored interventions, ensuring that every community fully benefits from vaccination programs

## Introduction

Vaccination is one of the most effective public health interventions, significantly reducing mortality and morbidity from vaccine preventable diseases (VPDs) ^1, 2^. Between 2000 and 2019, vaccination efforts in 98 low and middle-income countries (LMICs) averted about 37 million deaths, with substantial reductions among children under 5 years ^3^. Since 2010, new vaccines have been introduced in LMICs, in addition to the original Expanded Immunization Program (EPI) vaccines, such as pneumococcal, rotavirus, and human papillomavirus (HPV) vaccines ^4^. Forecasts for vaccine use in LMICs project about 17.7 million deaths will be averted in children under five years with improvement in vaccine coverage by 2030 ^5^. Beyond health impact, vaccines are cost-effective, saving about $350 billion in illness-related costs and generating about $500 billion in productivity gains, between 2011 and 2020 ^6^. Despite this impact, vaccination benefits are not equally shared across communities. Disparities persist due to variations in vaccine coverage between and within countries, with the gaps worsened by COVID-19 interruptions and existing health disparities. For instance, the number of “zero-dose” children increased from 12.9 million in 2019 to 14.3 million in 2021 ^7^, highlighting gaps in routine immunisation programs. Furthermore, some existing vaccines do not offer the same protection across regions; for instance Bacillus Calmette- Guerin (BCG) vaccine provided almost 100% protection against tuberculosis among UK school children infants, but achieved only 50% efficacy among Malawi adolescents ^8^. Similar patterns are observed with other vaccines such as rotavirus, polio, and Hepatitis B ^9, 10^. This variation in vaccine induced immune response may be influenced by individual characteristics such as age and nutrition status as well as environmental factors such as exposure to infections ^11^. Social, structural, and biological factors tend to interact, making some communities and individuals particularly vulnerable to suboptimal vaccine impact. The term vulnerability has been widely used across diverse domains, including disaster management, climate change, and infectious diseases to refer to poor health outcomes, risk or danger ^12– 14^. However, its definition remains ambiguous without context. This study defines vulnerability in the context of vaccination as the increased likelihood of communities or individuals experiencing suboptimal vaccination benefits. Understanding how structural, social, and biological factors interplay to impair vaccine impact is crucial for designing integrated strategies to address these challenges. The NIHR Global Health Research Group on Vaccines for Vulnerable people in Africa (VAnguard) ^15^ study was designed to address this gap. To inform the VAnguard study, a CVIVI that integrates data on structural, social, and biological factors that may impair vaccine impact was developed. By understanding the range and interplay of the underlying factors that contribute vulnerability to reduced vaccine impact, the CVIVI provides insights into specific challenges faced by communities. The information can be utilized to identify geographical areas at a greater risk of experience suboptimal vaccine impact.

## Methods

### Patient and Public Involvement

This study involves a secondary analysis of existing data. Patients and/or Public were not involved in research design, conduct, recruitment and dissemination plans.

### Vulnerability assessment framework

**Figure 1.**
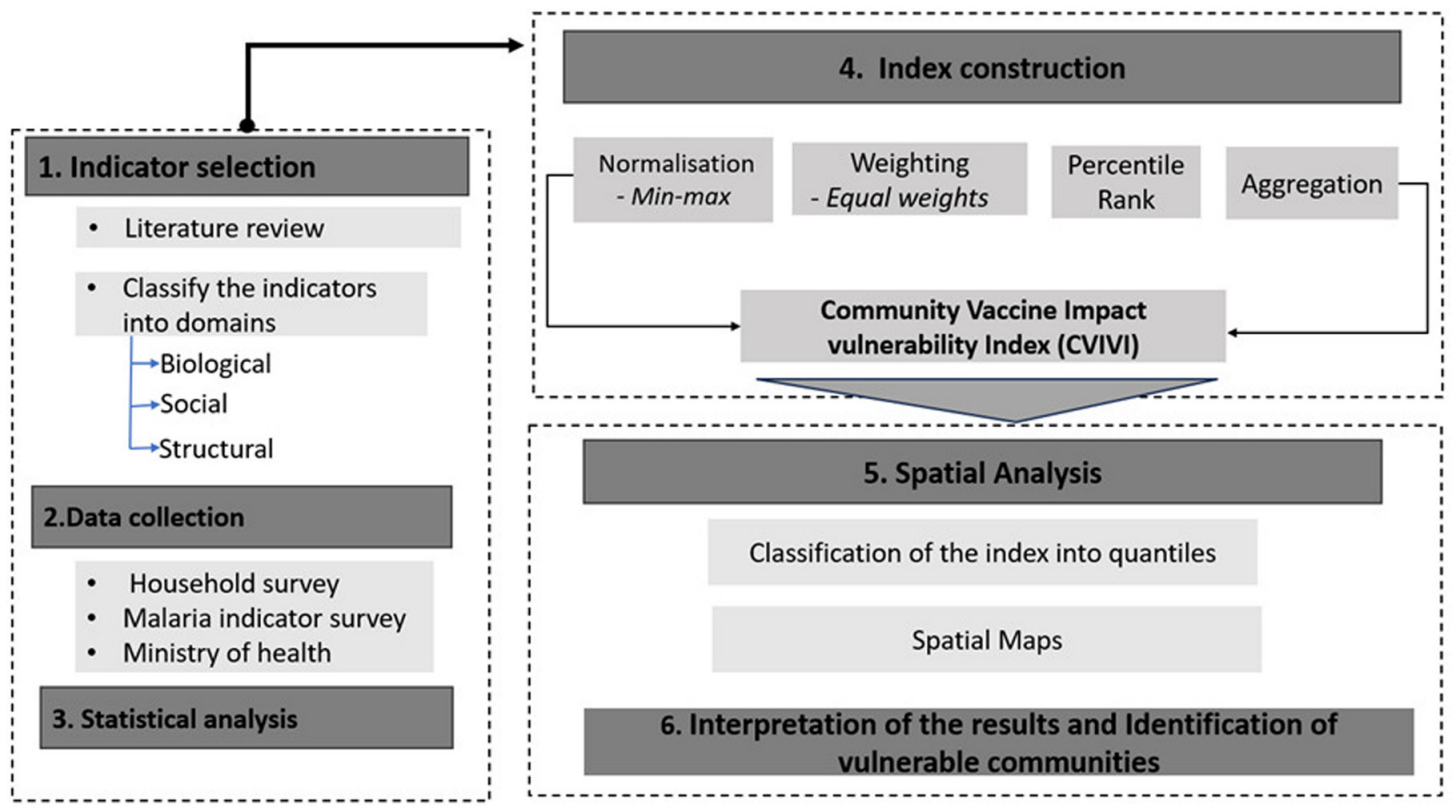
Workflow for assessing community vaccine impact vulnerability.

The Community Vaccine Impact Vulnerability Index (CVIVI) was developed and implemented following a structured process ( Figure 1), comprising six key aspects: (1) indicator selection, (2) data collection, (3) descriptive correlation analysis, (4) index construction, (5) spatial analysis, and (6) identification of vulnerable communities in Uganda and Kenya. 

### Indicator selection

Indicators were selected based on three criteria (i) the indicator’s relevance evidenced by literature review on vaccine immune response and uptake; (ii) data availability, and (iii) prevalence of the indicator in Uganda and Kenya. Based on these criteria, we selected 16 indicators, categorised into three domains: structural (seven indicators), social (six indicators), and biological (three indicators). These indicators reflect multidimensional factors that have each been demonstrated to influence vaccine impact. Table 1 shows the vulnerability domains, indicators used, and information on the data sources.

**Table 1.** Indicators used to define vaccine impact vulnerability.

Domain	Indicators	Definition	Source
Biological	Stunting	Percentage of children under five years who are stunted (greater than 2SD below the median height for age).	KDHS, 2022 ^23^, UDHS, 2016 ^22^
	Anemia	Percentage of children aged 6 months to 14 years who are moderately-severely anemic (low hemoglobin levels < 8 gldl)	Kenya MIS, 2020 ^49^, UDHS, 2016 ^22^
	Malaria	Percentage of children aged 6 months to 14 years who tested positive for malaria by rapid diagnostic test	Kenya MIS, 2020 ^49^, MOH, Uganda (2022)
	Helminths	Maximum point prevalence of schistosomiasis and soil transmitted helminth infections	Global Atlas of Helminth Infections ^50^
Structural	Distance to the nearest healthcare facility	Percentage of women aged 15–49 years who reported they faced a problem of long distance to the health care facilities	KDHS, 2022 ^23^ UDHS, 2016 ^22^
	Postnatal care	Percentage of live births (newborns) aged 12–23 months who didn’t receive postnatal check within 2 months after birth.	KDHS, 2022 ^23^ UDHS, 2016 ^22^
	Health Insurance	Percentage of households with no specific type of health insurance (National Health insurance fund, Private or community based)	KDHS, 2022 ^23^, UDHS, 2016 ^22^
	Place of delivery	Percentage of live births who were not delivered at a health facility	KDHS, 2022 ^23^ UDHS, 2016 ^22^
	Immunisation cards	Percentage of children 12–23 months who didn’t have vaccination card.	KDHS, 2022 ^23^, UDHS, 2016 ^22^
	Rural population	Percentage of households living in rural areas	KDHS, 2022 ^23^, UDHS, 2016 ^22^
Social	Household wealth	Proportion of children aged 12–23 months born to poorer/poorest households (according to the DHS wealth quintile classification)	Kenya MIS, 2020 ^49^ UDHS, 2016 ^22^
	Maternal education	Percentage of women with low level of education (either primary or no education)	Kenya MIS, 2020 ^49^, UDHS, 2016 ^22^
	Access to mass media	Percentage of women who had no access to specific media (newspaper, radio, TV) at least once in a week.	KDHS, 2022 ^23^, UDHS, 2016 ^22^
	Access to safe and clean water	Proportion of households without access to improved water sources	Kenya MIS, 2020 ^49^, UDHS, 2016 ^22^
	Housing conditions	Percentage of households living in informal dwellings	KCHSP, 2020 ^51^ UNPS, 2018 ^52^
	Poor sanitation facilities	Percentage of households with unimproved sanitation facilities	Kenya MIS, 2020 ^49^, UDHS, 2016 ^22^
	Transport means	Percentage of households with only non-motorised means of transport to the nearest health facility. *Non-motorised includes animal-drawn cart, bicycle, boat without a motor and walking.*	Kenya MIS, 2020 ^49^, UDHS, 2016 ^2^

### Biological vulnerability

Biological vulnerability measures susceptibility of individuals or populations to experiencing suboptimal vaccine induced immune responses ^16^. Individuals who are biologically vulnerable may exhibit shorter duration of protection or weakened immune responses, leading to reduced vaccine efficacy and increased susceptibility to VPDs. Previous studies, including a meta-analysis of the effects of infections ^17^, a review of nutritional factors ^18^ and a comprehensive review ^11^, have investigated factors that influence vaccine immunogenicity. In this study, we focus on modifiable biological factors common in African settings such as malnutrition, and parasitic infections, for which data are readily available.

***Malnutrition***

Malnutrition is a condition where a person`s nutrients or energy levels is deficient, excessive or imbalanced ^19^. It can manifest in different forms including underweight, overweight and micronutrient deficiencies (lack of important vitamins and trace minerals) ^19^. For purposes of this work, the focus is on common population level measure of malnutrition in Africa, specifically stunting ( a measure of underweight), and Anemia prevalence, form of iron deficiency. Malnutrition accounts for nearly 45% of child mortality in Africa ^20^. Stunting affects approximately 165 million children under five in Africa ^21^, with 26% ^22^ and 29% ^23^ prevalence in Uganda and Kenya, respectively. Nearly half (42.6%) ^23^ of the children in Kenya are anemic, compared to a lower prevalence of 29.4% ^22^ in Uganda. Malnutrition is likely to lead to immune deficiencies, which may adversely affect the quality of vaccine immune responses. For instance, a review study found out that, malnourished children tend to exhibit lower sero-protection and reduced efficacy for measles and rotavirus vaccines; however, data regarding other vaccines such as BCG and Hepatitis B remains inconcuslive ^24^. Furthermore, iron deficient children at the time of vaccination in Kenya showed reduced vaccine response to diphtheria, pertussis, and measles vaccines ^25^.

***Exposure to infections***

Parasitic infections such as helminths, malaria, and cytomegalovirus (CMV) are associated with impaired vaccine responses ^17, 26, 27^. Many African populations, particularly young children and pregnant mothers, are heavily exposed to these infections, due to poor access to clean water, inadequate sanitation facilities, poor housing conditions, and high levels of poverty ^28^. Helminths are highly prevalent in many African countries. For instance schistosomiasis prevalence among districts in Uganda ranges from 7.2% to 88.6% ^29^ and 2.1% to 18% among Kenyan preschool children ^28^. Helminth infections stimulate the production of regulatory T cells, which suppress inflammation and modulate the immune system to tolerate the parasite in the host’s body. This mechanism can weaken the body’s ability to mount strong immune responses to vaccines ^16, 17, 30, 31^. Additionally, chronic parasitic infections, like soil-transmitted helminths, are associated with stunted growth, anemia and micronutrients deficiencies, impacting immune function and vaccine effectiveness ^32, 33^. Similarly, malaria has been shown to reduce antibody production and long lasting immunity through immune dysregulation and immunosuppression, for instance a study in Uganda found decreased measles vaccine antibody responses in malaria exposed pregnant mothers and children, with similar results reported for BCG, tetanus, and pneumococcal vaccines ^34– 37^.

Therefore, based on the availability of data in Uganda and Kenya, the following indicators of biological vulnerability were chosen, prevalence of stunting, prevalence of anemia, and prevalence of malaria ( Table 1).

### Structural vulnerability

The social vulnerability domain includes social, cultural, and economic factors influencing vaccine access and acceptance. Several individual factors contribute to low vaccination rates in some parts of Africa, with maternal education and income levels being the most significant ^38– 40^. In Uganda, 54% ^22^ of women have low education levels, compared to 49% in Kenya ^23^, limiting their knowledge on benefits of vaccines, vaccination schedules, and potential risks of VPDs. This may result in vaccine hesitancy and poor decision making. Low income also affects vaccine uptake due to financial barriers such as transportation costs to the immunisation centers even when vaccines are free. Community related factors such as access to mass media, poor living conditions, also play a critical role in influencing vaccine impact ^41^. Limited access to reliable media sources can amplify misinformation and vaccine hesitancy, as seen with COVID-19 vaccine skepticism ^42– 44^. Poor living conditions such as poor housing structure, lack of clean water, and poor sanitation facilities, may negatively mediate the biological factors that influence vaccine efficacy. These conditions are prevalent in African countries, for instance, In Uganda, 24% of households had structures with poor roofing materials and about 21% lacked improved water sources in 2019. In Kenya, about 55% of the population lives in informal settlements ^45^. Such conditions facilitate disease transmission (e.g. tuberculosis, COVID-19, and malaria) ^46, 47^ and increase exposure to pathogens through contaminated food and water, hindering vaccine efficacy. For example, a trial among Zimbabwean infants showed that infants exposed to poor water, sanitation and hygiene (WASH) had reduced immune responses to rotavirus, evidenced by lower antibody levels and reduced seroconversion rates ^48^.

The following indicators of social vulnerability were therefore chosen: demographic factors, community factors such as poor living conditions, limited access to mass media, and possession of non-motorized means of transport ( Table 1).

### Structural vulnerability

Structural vulnerability includes physical, logistical, institutional, and policy related conditions that can affect delivery, distribution, accessibility, and quality of the immunisation services ^53– 55^. These factors are often external to the individual and operate at various levels including community, healthcare system, and national levels. Examples include: healthcare infrastructure, staff and training, policies and governance, and supply chain and logistics ^53– 55^. These factors may contribute to reduced vaccine impact by creating barriers to vaccine access and uptake. For instance, many African countries, vaccine availability is still a challenge in low income and middle countries. In Kenya, about 62.7% of the health facilities in Tana River County reported routine vaccine shortages in 2020 ^56^. Similarly, in Homia Uganda, facilities also experienced vaccine stockouts and inadequaties ^55^. The lack of vaccines at the facilities has been significantly associated with low vaccine coverage. For example in Nigeria, mothers reported making multiple visits to the health facilities on several occasions which was costly and time consuming and didn’t find the vaccines there, which may discourages and likely lead to incomplete immunisation for their children ^53^. Furthermore, geographic location including proximity to the nearest health facility, transportations and availability of reliable transport means were significant with a child being immunized ^53– 55^. For instance, in Turkana, Kenya, 38.6% of the households reported that their travel time to the nearest health facility was greater than two hours ^23^. This may account for the relatively low vaccine coverage in Turkana of, about 60%. Similarly, findings from a qualitative study revealed that the key structural factors facilitating uptake of COVID-19 vaccine among the elder persons were long distances to the vaccination sites, vaccine stockouts, and long waiting lines at the vaccination centres ^57^. Thus, the following indicators of structural vulnerability were chosen distance to the healthcare facilities, access to postnatal care services, health insurance coverage, place of delivery, possession of immunization cards, and rural population ( Table 1).

### Data collection

Data on the immunization program performance in Uganda for a period of 12 months from January to December 2022 were obtained from the Ministry of Health (MoH). Vaccine coverage was defined as the proportion of children under one year who have received three basic vaccines: first dose of measles (MR1), first (DPT1) and third (DPT3) dose of pentavalent vaccine.

Vaccine coverage data was obtained for all 146 districts, including the new districts. For Kenya, immunization data was obtained from the Kenya Demographic Health survey (KDHS,2022) ^23^. Vaccine coverage was defined as the proportion of children aged 12–23 months receiving all the basic antigens; BCG vaccine; three doses of polio (OPV, IPV); three doses of DTwP-Hib-HepB; single dose of Measles-Rubella (MR). The variation in the definitions for vaccine coverage was due to the different data sources and the data available for specific vaccines. In Uganda, vaccine coverage was calculated using administrative data collected as part of the Red Every District (RED) strategy, used to evaluate immunisation performance for each district. This approach focuses on coverage for measles (MR1), DPT1 and DPT3, that are routine tracked through health records and are available for most districts. On the other hand, in Kenya, household survey data was used, which includes a wide range of antigens. Data for risk factors for impaired vaccine impact was obtained from household surveys, including demographic health survey (DHS) conducted in Uganda (2016) and Kenya (2022). Data on malaria prevalence was obtained from the Malaria Indicator survey (MIS) reports and ministry of health ( Table 1). Newly created districts in Uganda (beyond the 123 defined in the 2016 UDHS) were excluded due to incomplete data. In the construction of the index, we excluded helminths prevalence as the data was not consistently available for all counties and districts. The data was then linked to the shape files for spatial analysis.

### Index construction

The CVIVI was constructed in four steps, namely, normalization, percentile rank calculation, weighting, and aggregation ( Figure 1).

### Normalization

Indicator values were scaled between 0 (least vulnerable) to 1 (most vulnerable) using the max-min approach ^58, 59^ to standardize the disparate data scales.


$$y_{in}=\frac{X_{in}-\text{min}(X_{in})}{\text{max}(X_{in})-\text{min}(X_{in})}$$

Where *y _in_* is the normalized indicator and *X _in_* is the indicator value 

### Percentile rank calculation

The percentile rank for all the selected indicators presented in Table 1 was calculated. The percentile rank methodology has been commonly employed in defining vulnerability indices related to infectious diseases such as COVID-19 ^60^ and climate change ^61^. Each indicator was ranked in ascending order such that higher values indicate greater hypothesised vulnerability. Districts or counties were assigned ranks, and the percentile rank for all the selected indicators described in Table 1 was computed using the formula:


$$Pr_{ij}=(r_{ij}-1)/(N_{j}-1)$$

where *r _ij_* is rank of indicator *j*, in district/ county *i*, *N _j_* is the number of districts/counties with indicator j and *Pr _ij_* represents the percentile rank of indictor *j*, in district/county *i*. The percentile rank is a statistical measure ranking each data point in relation to the full dataset (for instance 40th percentile represents the value below which 40% of the data falls). In this context, higher percentile rank values denote higher vulnerability ( *Pr _ij_* = 1.0), while lower values represent lower vulnerability ( *Pr _ij_* = 0.0).

### Weighting

The final vulnerability index calculation is determined by the choice of the weights, and there are several ways to determine these weights including statistical methods such as principal component analysis (PCA), factor analysis, equal weights, and participatory approaches such as Analytic Hierarchy Process (AHP) ^58, 59^. To avoid bias and for simplicity, an equal weight approach was adopted where indicators were equally weighted within each domain and each domain was then given equal weights such that biological, social and structural domains each contribute equally to the composite vulnerability index. The decision to apply equal weights stemmed from the recognition that domains inherently comprise of different numbers of indicators. A similar approach has been applied in index construction of established indices such as the Surgo Foundation Community COVID-19 vulnerability index ^62^ as well as the Centre for Disease Control and Prevention (CDC) social vulnerability index ^63^.

### Aggregation

The domain vulnerability of each district/county was obtained by summing the percentile ranks for all indicators in each specific domain, given as


$$DV_{ik}=\frac{\sum_{j=1}^{n}Pr_{ij}}{n}$$

where *k* represents the number of domains i.e. *k* = 1, 2, 3 as shown in Table 1; *DV _ik_* represents the vulnerability value of district/county *i* computed based on indictors in domain, *k*, and *n* is the number of indicators in each domain. The Community Vaccine Impact Vulnerability Index, CVIVI _i_ for each district/county was calculated as the average of domain-specific scores as follows:


$$CVIVI_{i}=\left(1/3\right)*\sum_{k}DV_{ik}$$

For easy interpretation of the results, the developed index values were normalized in the range of zero to one where zero indicates least vulnerability and 1 indicates very risk to reduced vaccine impact. It’s important to note that the CVIVI measures the relative levels of vulnerability to reduced vaccine impact among districts or counties, which means that an index of value 0.0 doesn’t imply absence of vulnerability to reduced vaccine impact within the district or county. 

### Statistical analysis

Pairwise correlation analysis was performed to describe the relationships between each pair of indicators, and between the index and vaccine coverage. Pearson’s correlation coefficients were reported since the underlying data used were normally distributed.

### Spatial analysis

The spatial primary units of analysis were districts in Uganda and counties in Kenya since they were the smallest administrative unit available with complete data. Choropleth maps were generated using QGIS to visualise geographical patterns at the district or county level. The CVIVI scores were classified into five quintiles, ranging from least to most vulnerable. Bivariate maps were used to overlay vaccine coverage and vulnerability scores, allowing for a visual assessment of correlations between vaccine coverage and domain specific vulnerabilities. Some of the vaccine coverage estimates in Uganda exceeded 100%, and these were rounded down to 100% for spatial mapping. To generate the bivariate maps, data on vaccine coverage and the CVIVI scores were categorized into tertiles (3 quantiles: high, moderate, and low).

## Results

### Relationship between vulnerability indicators

**Figure 2.**
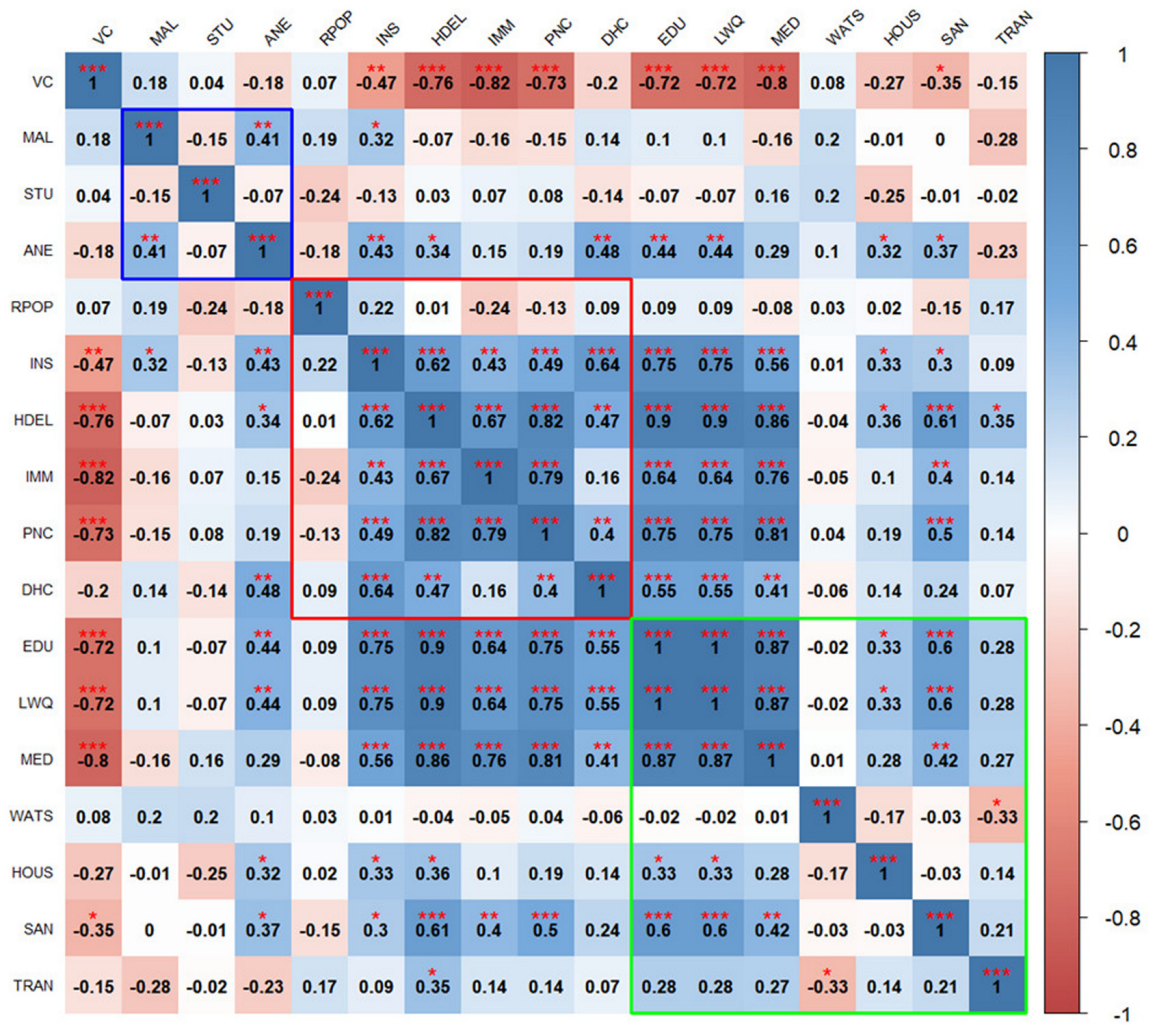
Pairwise correlations between vulnerability indicators in Kenya. Blue outline represents biological factors, red outline represents structural factors, and green outline represents social factors. Asterisks indicate level of significance: ***p value < 0.001, **p value < 0.01, *p value < 0.05.

**Figure 3.**
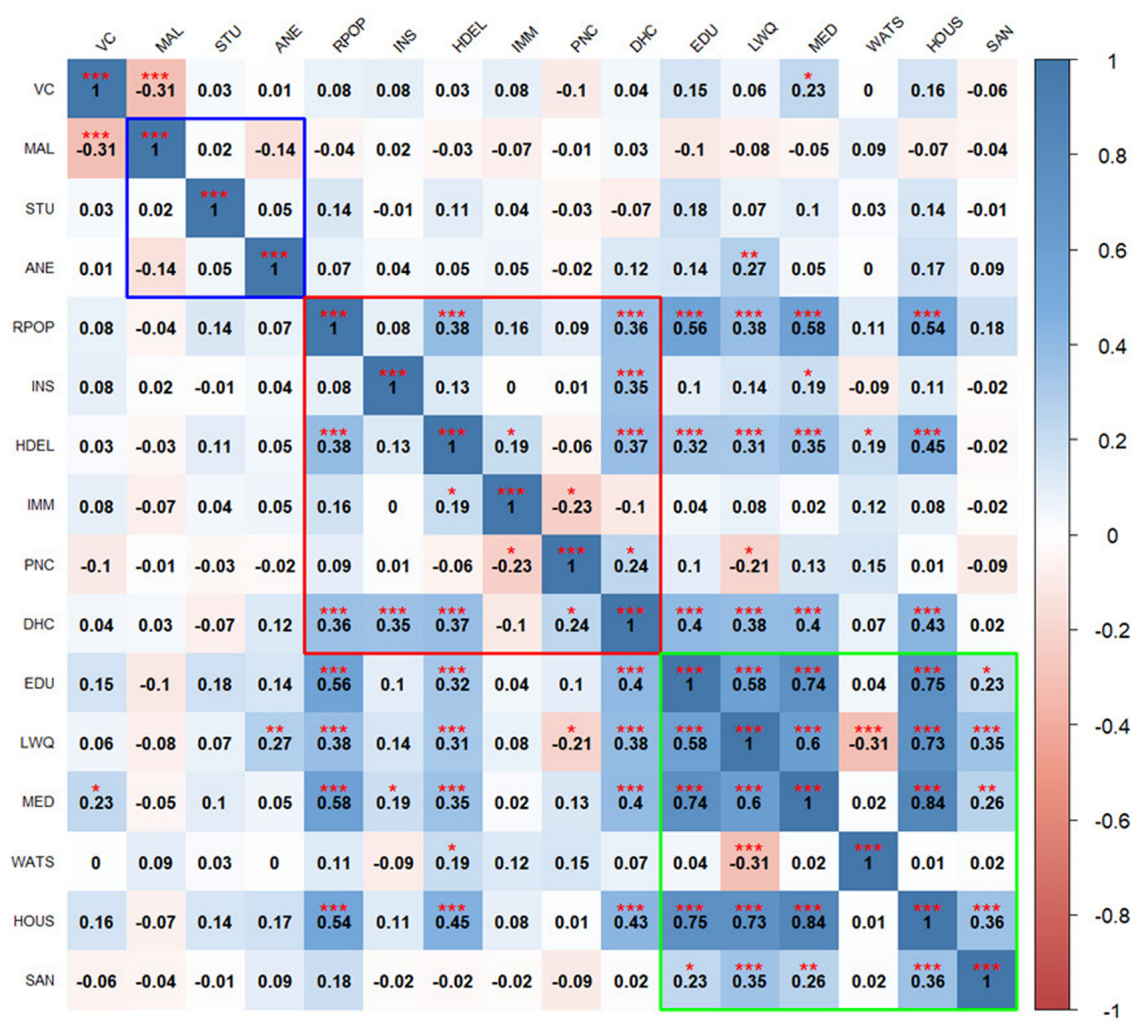
Pairwise correlation between indicators in Uganda. Blue outline represents biological factors, red outline represents structural factors, and green outline represents social factors. Asterisks indicate level of significance: ***p value < 0.001, **p value < 0.01, *p value < 0.05. The acronyms used in the figure 3 and Figure 4 are as follows: **VC** (Vaccine Coverage), **MAL** (Malaria prevalence), **STU** (Stunting prevalence), **ANE** (Anemia prevalence), **RPOP** (Rural population), **INS** (Insurance coverage), **HDEL** (Home delivery), **IMM** (No immunization card), **PNC** (No postnatal care for newborns), **DHC** (Long distance to nearby health facility), **EDU** (Low mother’s education level), **LWQ** (Low wealth quintile), **MED** (Limited access to mass media), **WATS** (Access to unimproved water sources), **HOUS** (Poor housing structures), **SAN** (Access to unimproved sanitation facilities), and **TRAN** (Ownership of non-motorized transport means).

The correlation heatmaps in Figure 2 and Figure 3 illustrate the pairwise relationships between vulnerability indicators themselves, as well as their relationship with vaccine coverage in Kenya and Uganda, respectively. Each cell represents the correlation coefficient between two variables, with positive correlations shown in shades of blue and negative correlations in shades of red.

**Figure 4.**
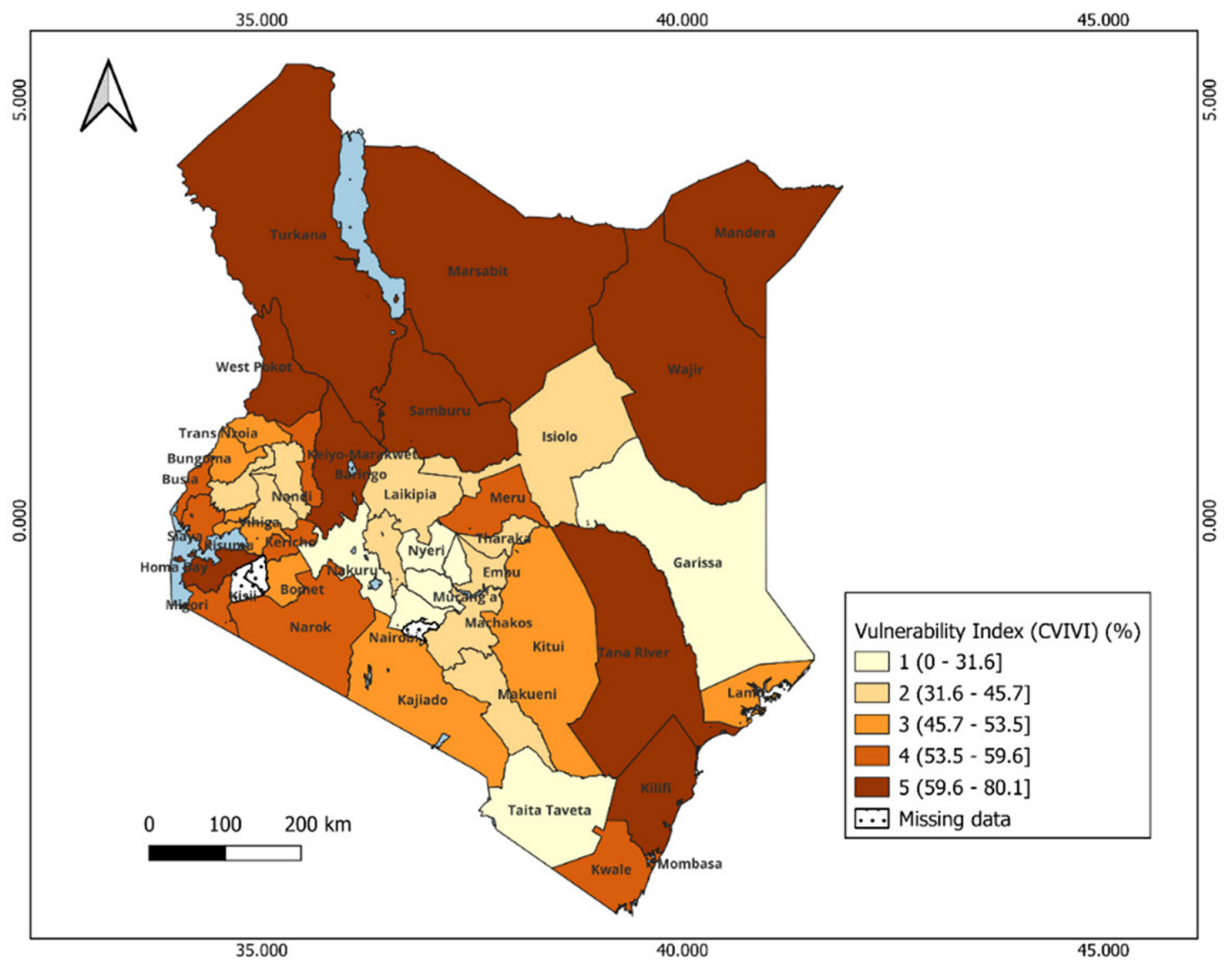
Spatial Distribution of CVIVI scores across counties in Kenya. Dark colors represent high vulnerability, and light colors correspond to low score vulnerability. Scores are categorized by groups: 1= least vulnerability, 2 = less vulnerable, 3 = moderately vulnerable 4= More vulnerable, 5= most vulnerable.

In Kenya, there is significant negative correlation between vaccine coverage and structural factors such as high home-based deliveries, lack of immunization cards, no postnatal care for newborns, and social factors such as low maternal education, low family income, and limited access to media. No consistent patterns of correlation were seen between vaccine coverage and biological factors, with positive and negative correlations observed within this domain. For example, anemia is positively correlated with malaria, while stunting is negatively associated with malaria.

Biological indicators show weak or inconsistent correlations with social and structural indicators, though some notable relationships exist, for instance, higher anemia prevalence is linked to home deliveries and lack of insurance coverage. Structural indicators are positively correlated with each other except for the percentage of rural population. Structural factors significantly correlate with most social factors, particularly maternal education, income level, and access to media. Social indicators are strongly correlated with each other.

In Uganda, the findings show that vaccine coverage is negatively correlated with high malaria prevalence ( Figure 3). Also, vaccine coverage is poorly correlated with all the structural factors. Access to media stands out as the only social factor positively correlated with vaccine coverage.

Biological factors exhibit weak correlation with each other and with indicators in other domains, except, anemia prevalence, which correlates positively with low wealth quintile. Within the structural domain, few indicators show significant positive correlations with others such as rural population, home based deliveries, long distance to the health facilities showing positive weak correlations while lack of postnatal care exhibits negative correlations. Social factors are generally positively correlated with each other, except for limited access to improved source water. Rural population and home-based deliveries positively correlate with low maternal education, low income, limited access to media access and poor housing structures.

### Vulnerability to reduced vaccine impact and underlying factors

The index reveals significant geographical patterns in the vulnerability levels between and within each country, as shown in the maps ( Figure 4 and Figure 6) and Extended data, *Tables 1 & 2* in Kenya and Uganda, respectively. The dark colors indicate districts at a high risk of reduced vaccine impact, indicating that communities in these areas are less likely to benefit from vaccines. Bar plots 5 and 7 present the average estimates of key indicators across each vulnerability group, helping us identify the main factors contributing to vulnerability in each group. The contributing factors were those with relatively higher values as compared to other areas (see *Extended Data Tables 4 & 5*).

**Figure 5.**
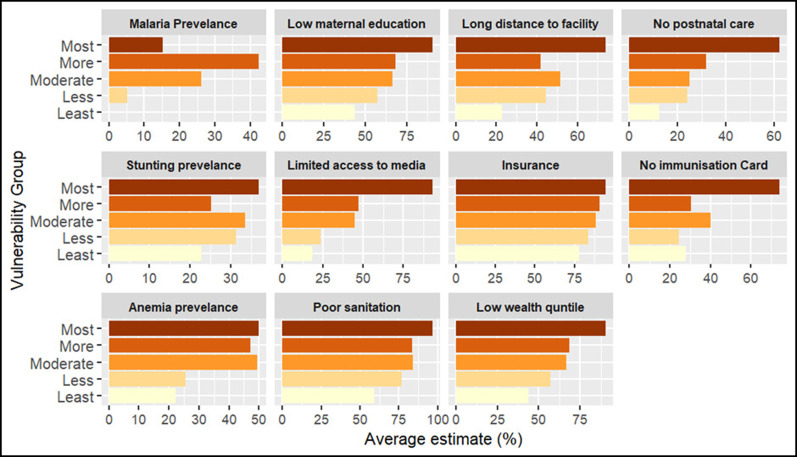
Percentage distribution of indicators across vulnerability groups in Kenya. The figure presents the average estimate of key indicators across vulnerability groups, from the most vulnerable (dark shades) to the least vulnerable (light shades). Each panel represents a specific indicator, illustrating its contribution to community vulnerability.

In Kenya, most vulnerable counties form clusters within specific regions. For instance, most vulnerable counties include Mandera, Turkana, Garissa in the eastern, West Pokot, in the western and Kilifi and Tana River in the southern region. These counties face a combination of social, structural and biological challenges such as the high prevalence of anemia, lack of immunisation cards, low maternal education, long distance to the near health facility, limited access to post-natal care services for newborns, low wealth quintile, and limited access to mass media ( Figure 5). Despite these challenges, malaria prevalence in these counties is relatively lower than compared to other counties (see *Extended data, Table 4*). Counties with moderate vulnerability are primarily located in the central and southern parts of Kenya. Examples of these counties include: Kwale, Meru, Kajiado, and Narok. Conversely, counties in the central region (e.g., Kiambu, Nyeri, Machakos) and Taita Taveta (south) show low vulnerability scores ( Figure 4). These counties benefit from better healthcare access and high levels of maternal education, which is likely to contribute to the low prevalence of biological indicators such as malaria and stunting (see *Extended data, Table 4*). Notably, health insurance coverage remains relatively low across all counties, regardless of their vulnerability level. Also, most of the households had poor access to unimproved and shared toilets.

**Figure 6.**
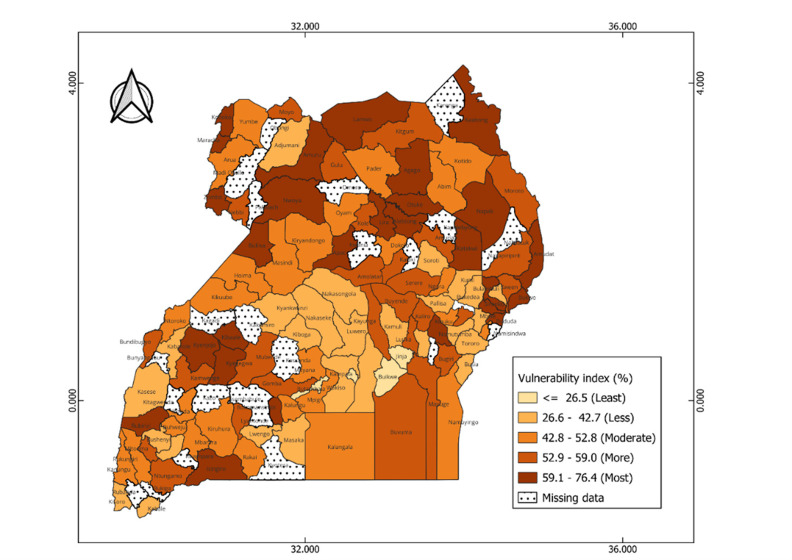
Estimates of the vulnerability index across districts in Uganda. The index scores are grouped onto 5 groups with the dark colors representing high vulnerability scores, and light colors correspond to low score vulnerability scores.

**Figure 7.**
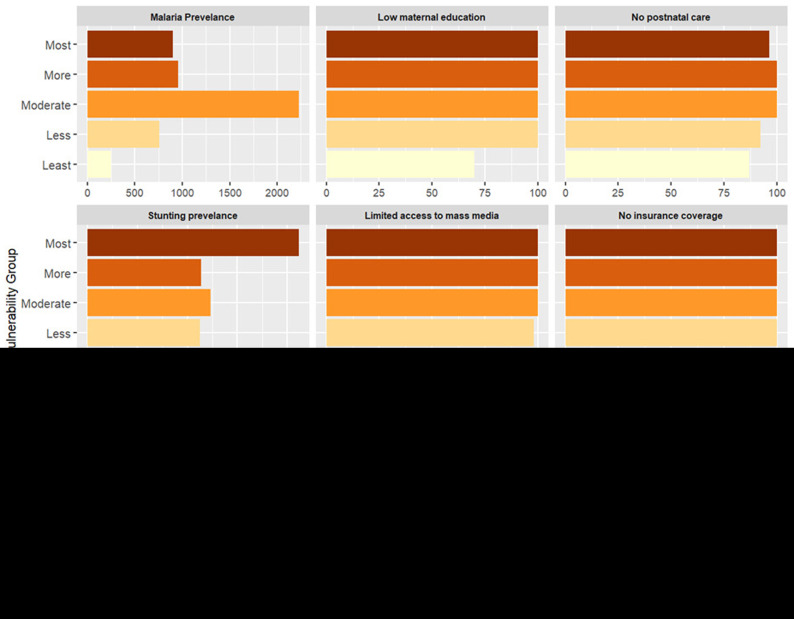
Percentage distribution of indicators across vulnerability groups in Uganda. The figure presents the average estimate of key indicators across vulnerability groups, from the most vulnerable (dark shades) to the least vulnerable (light shades). Each panel represents a specific indicator, illustrating its contribution to community vulnerability.

In Uganda, vulnerable districts are scattered across all regions. For instance, most vulnerable districts such as Amudat and lamwo are in the northern region, Buliisa and Kyenjojo in the western region, and Bulambuli and Bududa in the eastern region ( Figure 6). Communities in these districts are faced with significant challenges including high stunting prevalence, high levels of poverty, limited access to postnatal care services for newborns, low maternal education levels, and limited access to mass media ( Figure 7). Moderately vulnerable districts are also observed across different regions such as Kotido and Abim in the north, Namayingo and Ngora in the east, and Mpigi and Kayunga in the central. The least vulnerable districts are mainly concentrated in the central region such as Kampala, Buikwe, and Butambala. Despite their low vulnerability, these areas still face some challenges. For instance, Kampala, an urban district is characterized by high malaria incidence (218.5 cases per 1000 population), low insurance coverage (96.3%), limited access to postnatal care services (68.4%), and limited access to mass media by women (55.8%) (see *Extended data, Table 5*).

### Domain specfic vulnerability patterns

The overall CVIVI is a composite score that may obscure specific challenges. Districts or counties with low CVIVI scores may still exhibit high scores in at least one domain. Thus, disaggregating the index into structural, social and biological domain scores reveals distinct geographical patterns as shown in spatial maps in *Extended data, Figure 1 & 2* and *Extended data, Table 1 & 2*

### Social vulnerability score

In Kenya, social vulnerability is highest in northern and coastal counties ( *see Extended data, Figure 1- panel A*), driven by limited access to mass media, poor sanitation and low maternal education levels (see *Extended data, Table 5*). Mandera county exhibits the highest prevalence of these barriers. In Marsabit county, 33.4% of the households lack improved water sources, while 92.3% lack access to proper sanitation facilities. Moderately vulnerable counties, such as Isiolo, Kitui, and Busia have better access to mass media and improved water sources, balancing their overall vulnerabilities. Conversely, counties in central and southwest Kenya show low vulnerability due to higher maternal education levels, better media access and low poverty levels, though they struggle with inadequate sanitation facilities, with toilet facilities often shared.

In Uganda, the most socially vulnerable districts are concentrated in the northern region, with a few in the southwest (see *Extended data, Figure 2-panel D*) characterized by high poverty, low mothers' education, limited access to mass media, poor housing conditions, and unimproved sanitation facilities, despite better access to improved water sources. Districts in the Central region are least vulnerable, with relatively high-income levels and better infrastructures, though women still face challenges with limited media access.

### Structural vulnerability score

In Kenya, high structural vulnerability was observed among counties predominantly situated in northern and southeast Kenya ( *see Extended data, Figure 1-panel B*), driven by limited insurance coverage, reliance on non-motorized transportation, and limited access to postnatal care for newborns. Conversely, the less vulnerable counties like Kericho, Nandi, and Kajiado, are characterized by improved healthcare services, and improved means of transport. However, structural challenges, such as high rates of uninsured households (e.g. 82% in Nyeri), persisted even in less vulnerable communities (see *Extended data, Table 4*).

In Uganda, structural vulnerability was unevenly distributed, with the highest scores in the northern districts and a few districts in the southwest (see *Extended data, Figure 2-panel E*). These areas are predominantly rural, facing challenges such as low insurance coverage, home deliveries, and limited access to postnatal services. Strikingly like the Kenyan scenario, despite their vulnerability, these districts exhibit a higher proportion of children with immunization cards. Moderately vulnerable districts in central and southwestern Uganda (e.g. Hoima, Kikuube, and Masaka) exhibited mixed outcomes. While these areas demonstrate positive indicators like the high prevalence of health facility deliveries and access to postnatal care services, household insurance coverage remains a significant challenge. Urban districts like Kampala and Wakiso districts were the least vulnerable due to better access to healthcare services and widespread immunisation services, though gaps in access to postnatal services and insurance coverage persist.

### Biological vulnerability score

In Kenya, counties located in the coastal areas (e.g. Kilifi) and the southwest region (such as Turkana and Tana River) display high biological vulnerability due to high prevalence of stunting and anemia among children aged 6–35 months (see *Extended data, Figure 1-panel C*). Additionally, malaria prevalence is particularly high in some counties such as Busia and Kisumu, due to their low-lying and humid environment. The least vulnerable counties are in the central region of Kenya, such as Samburu, Baringo, and Kiambu, as well as Taita Taveta in the southern region of Kenya. In Uganda, the results show that different biological vulnerability levels are dispersed across the country ( *see Extended data, Figure 2-panel F*), emphasizing the heterogenous nature of the biological factors. Districts such as Koboko, Nakapiripit, and Lira situated in the northern region and water-body proximate districts such as Buliisa and Kalangala exhibit high vulnerability, primarily attributed to high prevalence of malnutrition, and high malaria prevalence, respectively.

### Heterogeneity in community vulnerability profiles

**Figure 8.**
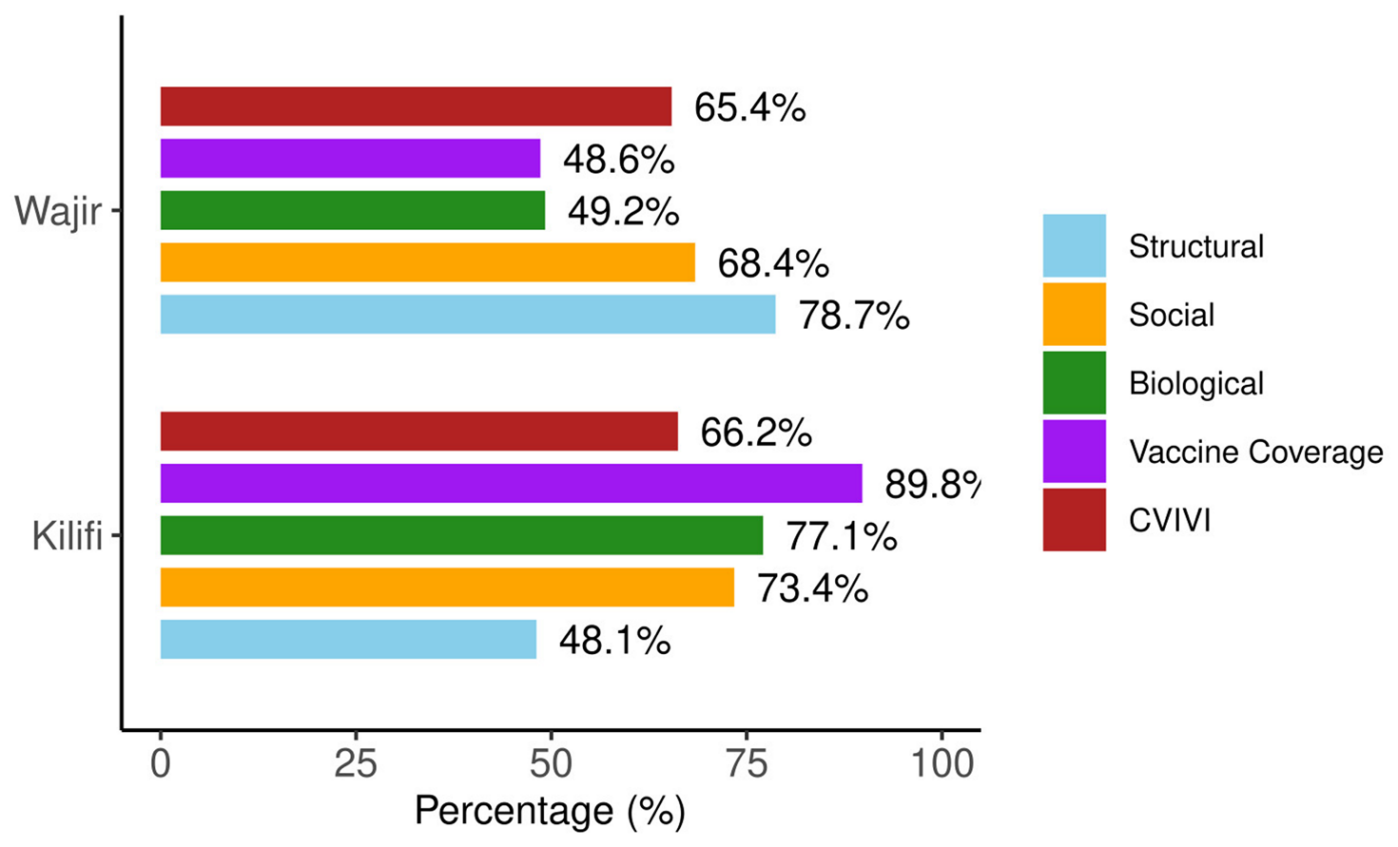
Overall and domain specific vulnerability scores for Wajir and Kilifi counties in Kenya.

Our analysis reveals that communities with identical overall vulnerability scores can exhibit markedly different vulnerability profiles across domains. To illustrate this, we compared two counties in Kenya: Wajir and Kilifi counties. These have nearly identical overall CVIVI scores (65.4% and 66.2%, respectively) but with different domain-specific vulnerability scores ( Figure 8).

 In Wajir, structural factors are primary drivers of vulnerability, with the county scoring 79 on this domain. Most households in Wajir reported facing long distances to the nearest health facilities, and 62.2% of women did not receive postnatal care for their newborn infants (see *Extended data* Table 4 ^64^). Wajir is one of the counties with low vaccine coverage (48.6%). On the other hand, biological and social factors are the main vulnerability drivers in Kilifi scoring 77% and 73%, respectively. Biological vulnerability is largely attributed to the high prevalence of anaemia among children (45%), which is relatively high when compared with other counties in Kenya. Social vulnerability is characterised by high poverty levels (69%), low maternal education (69%), and poor sanitation (80%). Kilifi exhibits a relatively high vaccine coverage (89.8%).

### Distribution of vaccine coverage in Uganda and Kenya

**Figure 9.**
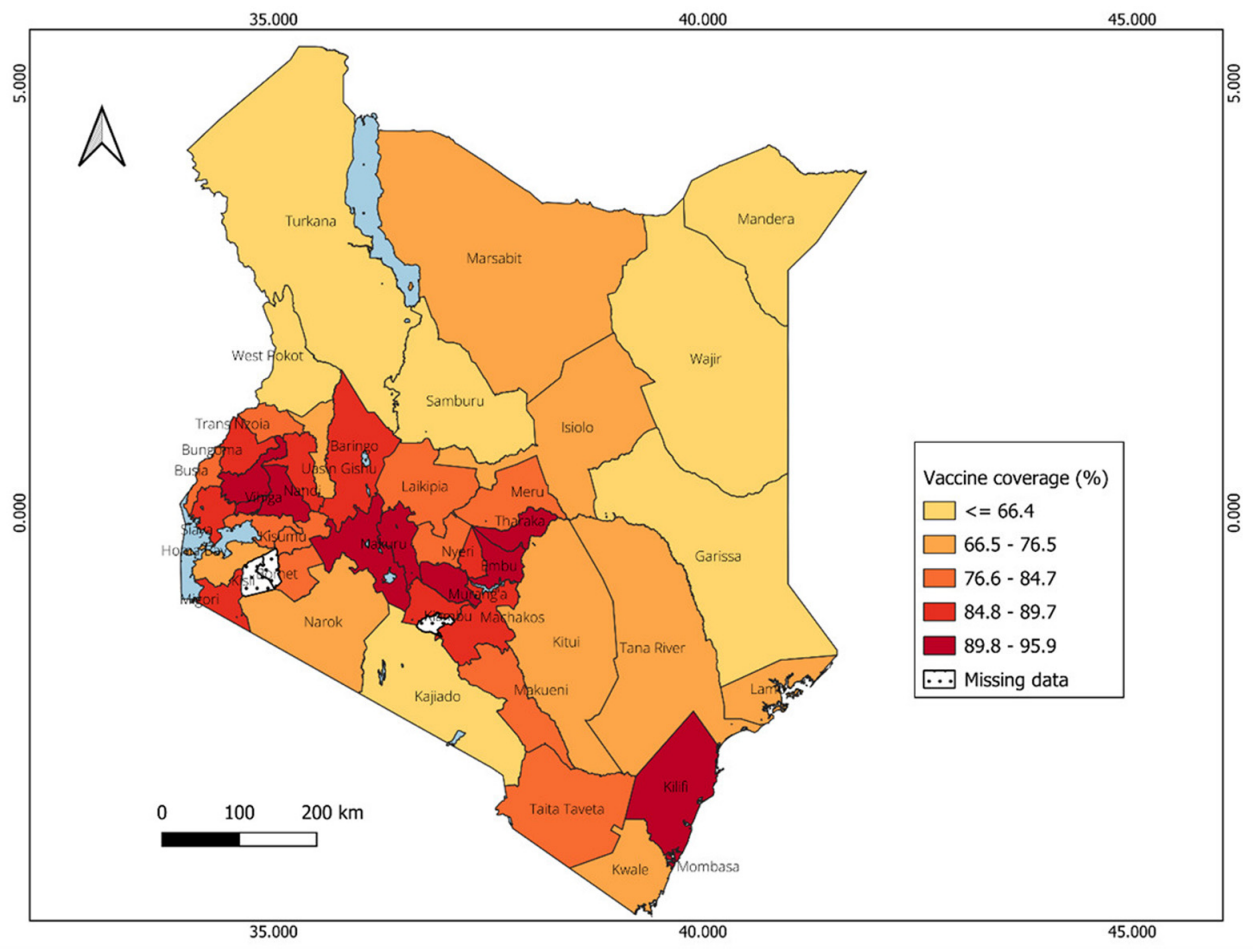
Spatial distribution of vaccine coverage across counties in Kenya.

In Kenya, significant variations in vaccine coverage are observed across counties. The lowest vaccine coverage is reported in counties boarding Somalia, such as Garissa and Mandera, ranging from 20% to 40% ( Figure 9). Counties with highest vaccine coverage are located mainly in the central region, with vaccine coverage estimates ranging from 79% to 96%, and Vihiga having the highest percentage (96%).

**Figure 10.**
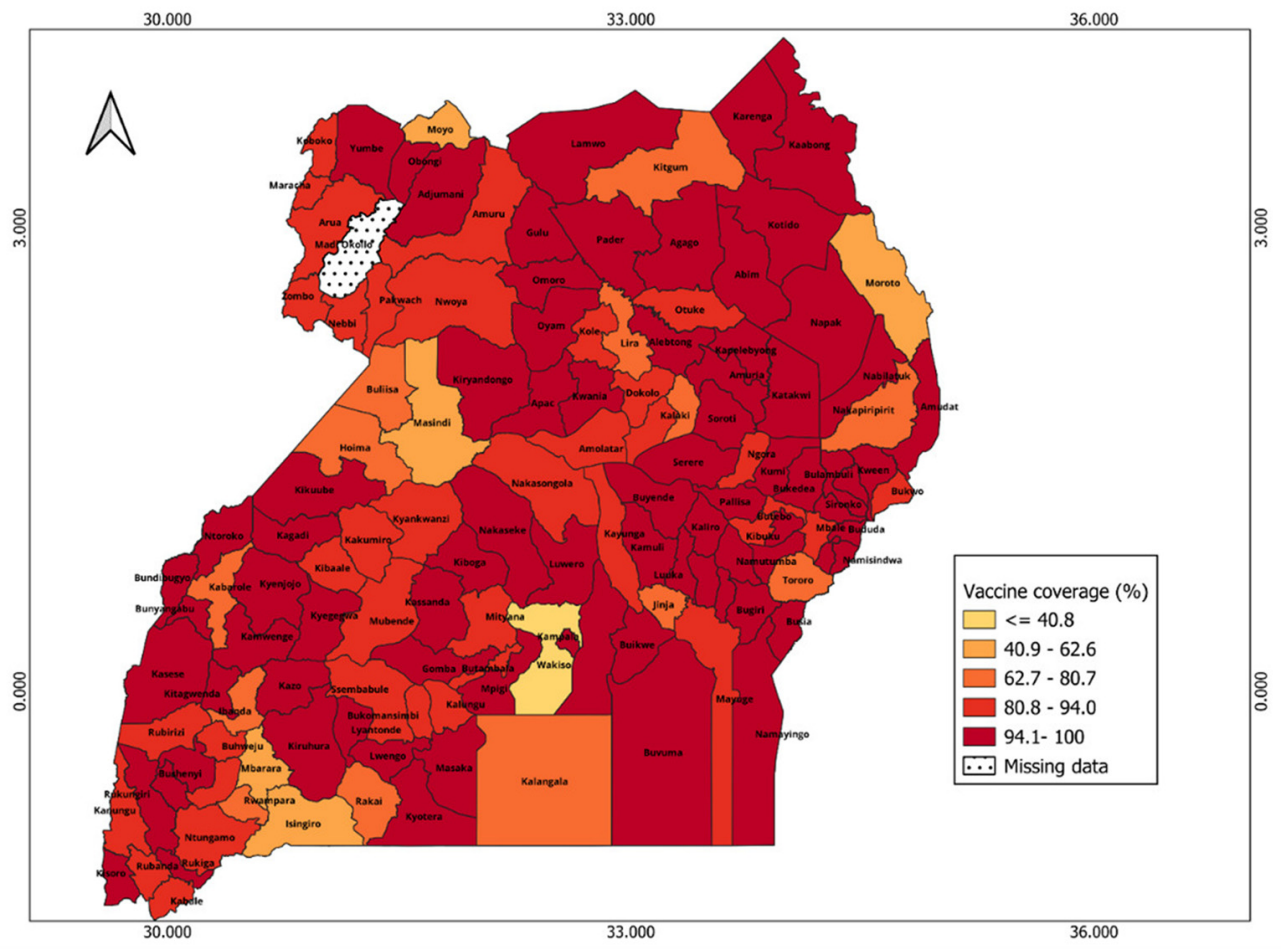
Spatial distribution of vaccine coverage across districts in Uganda.

In Uganda, many districts (72 out of 145) reported vaccine coverage greater than 100%, while only one district has missing data. The map ( Figure 10) shows less geographical heterogeneity in the spatial distribution of vaccine coverage across districts, especially when compared to Kenya. Most of the districts in the north and central regions have relatively high vaccine coverage except districts in hard-to-reach areas like Nakapirit (74.3%) and some rural districts like Buliisa (69.3%). Wakiso has the lowest vaccination coverage (40.8%).

***Correlation between vaccine coverage and vulnerability index***

**Figure 11.**
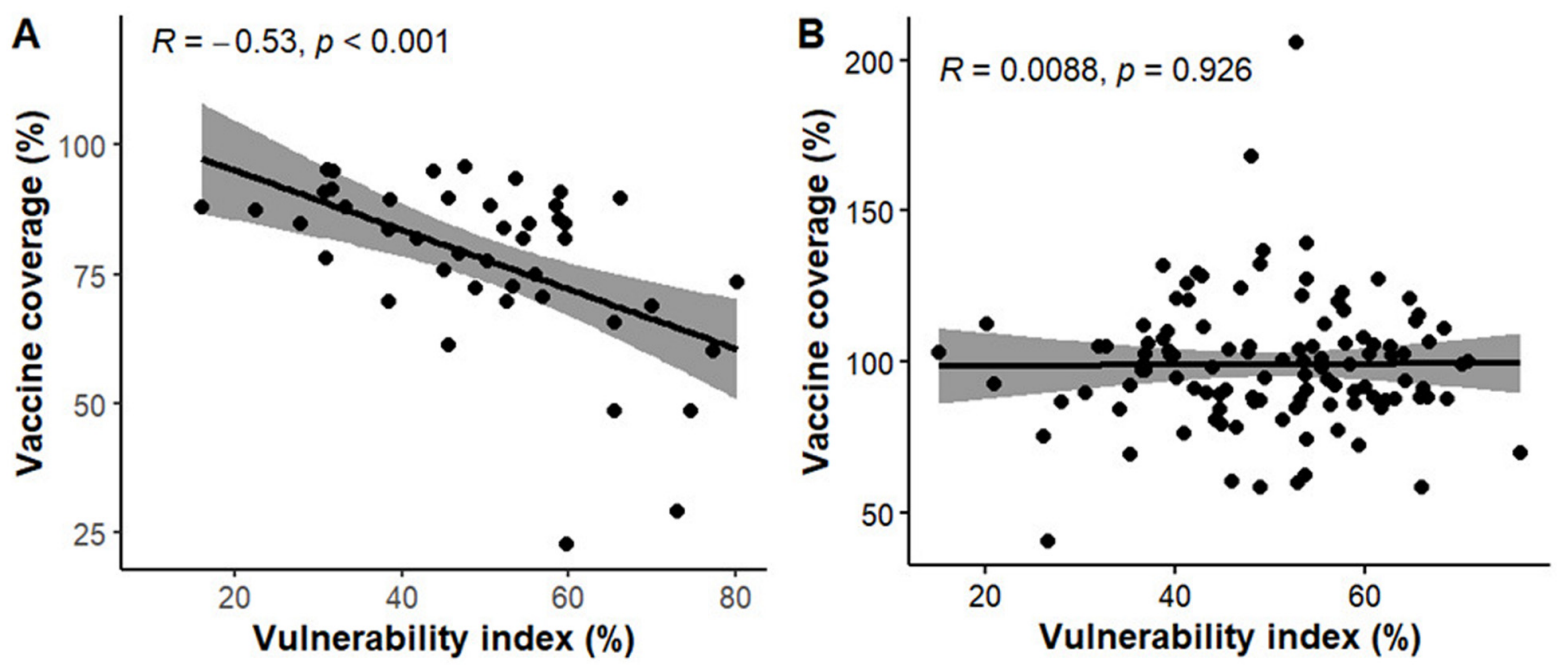
Correlation plots showing the relationship between vulnerability index scores and Vaccine coverage. Solid line shows linear regression fit while the shaded part shows the 95% confidence interval.

In Kenya, a negative correlation (R= -0.53, p < 0.001) is observed between vaccine coverage and CVIVI score ( Figure 11, panel A), indicating that counties with lower vaccine coverage tend to have higher CVIVI scores. These findings are illustrated in the bivariate map, which shows clustering of high vulnerability scores in areas with low vaccine coverage ( *see Extended data, Figure 3*). For example, northwest counties like Turkana, West Pokot, and Mandera, as well as coastal regions such as Tana River, exhibit both high CVIVI and low vaccine coverage. Conversely, counties in the central region demonstrate high vaccine coverage and low CVIVI scores. Interesting, Garissa, which had the lowest vaccine coverage also has the lowest CVIVI score, suggesting that the factors considered may not explain the low vaccine coverage in this county. In Uganda, there is no correlation between CVIVI and vaccine coverage (R = 0.009, p = 0.925) ( Figure 11, panel B). Bivariate maps ( *see Extended data, Figure 4*) highlight a small number of central districts, such as Kampala and Wakiso, which have low CVIVI scores and high vaccine coverage. Notably, Buliisa stands out as the only district with both high CVIVI and low vaccine coverage.

## Discussion

This study developed a community vulnerability index to identify communities in Uganda and Kenya at a risk of reduced vaccine impact and why, based on underlying community’s structural, social and biological factors. Our findings reveal distinct patterns in the geographic distribution of vulnerability within and between the two countries. In Kenya, different vulnerability levels were clustered in specific regions, with the most vulnerable counties mainly found in the northwest and southeast of Kenya including Turkana, Mandera, and West Polot counties. In Uganda, vulnerability was more scattered, with the most districts concentrated in the northeast (such as Amudat, Lamo) and southwest (such as Buliisa and Kyenjojo). These areas primarily comprise of rural communities, geographically isolated regions, and some have a significant number of refugees, particularly in northern Uganda ^65^. In addition, some of these vulnerable districts have experienced severe disease outbreaks for instance recent measles outbreak in Lamwo, Kasese and Amuru districts ^66^. The contributing factors to high vulnerabilities in these areas are shown to cut across various domains, demonstrating multiple interrelated factors may contribute to reduced vaccine impact ^67^. In both countries, the most prevalent structural factors were; long distance to the nearest health facility, and lack of postnatal care for newborns. Among social factors, low maternal education, and poor households were prominent, while for biological factors, the prevalence of stunting and anemia was relatively high. Additionally, challenges such as limited access to health insurance were highly prevalent even within less vulnerable communities. Although previous studies have shown associations between these risk factors and vaccination effectiveness, this work contributes to the existing literature by highlighting factors that co-exist in specific districts/counties ^38, 68^. These findings highlights the need for cross-sector collaborations between health, education, and infrastructure sectors to improve vaccine benefits and promote health equity in these vulnerable communities. Our analysis further reveals heterogenous community vulnerability profiles even with identical overall vulnerability scores, with each district/ county vulnerable at least in one domain. This suggests that different communities face unique challenges implying that universal solutions may not effectively address the health disparities associated with these risk factors within these communities, potentially leading to impaired vaccine impact. Tailored, context-specific interventions are crucial to address distinct challenges faced by each community. For instance, in vulnerable counties like Wajir, Kenya, where vaccine access is likely to be hindered by long distances, strategies such as mobile vaccination clinics and community outreach programs could bridge access gaps. In contrast, in counties such as Kilifi, where biological and social vulnerabilities are high, integrated interventions might focus on poverty alleviation, maternal education, and nutritional support to reduce anemia prevalence. The findings also demonstrate negative correlation between the vulnerability index and vaccine coverage, particularly in Kenya where heterogeneity in vaccine coverage was observed. However, in Uganda, despite notable geographic disparities in the index, no correlation between the vulnerability index was observed. This is likely due to the uniform distribution of the vaccine coverage estimates across districts as well as data quality issues ^69^. This highlights that relying solely on vaccine coverage estimates to evaluate vaccine impact vulnerability and identify vulnerable communities may not be an effective approach. Nevertheless, our findings suggest that the CVIVI has the potential to identify vulnerable communities in situations where poor quality or unreliable data on vaccine coverage exists.

### Strengths and limitations

This study has some limitations. The study relied on existing datasets that may have gaps, inconsistencies and outdated information, particularly for data from 2016 Uganda Demographic Health survey. Inconsistencies in the vaccine coverage data for Uganda were observed, with some district-level estimates (72 out of 145) exceeding 100% due to denominator issues ^69^. Additionally, differences in the definition of vaccine coverage used for Uganda and Kenya may also affect the comparability of vaccine findings between the two countries. Our analysis was based on aggregated data at district and county levels, which may mask heterogeneity within these large administrative units. While the index was useful for identifying communities, it doesn’t fully capture the interplay between social, structural and biological factor that contributes to reduced vaccine impact. Future work should consider conducting more fine-scale analyses such as household surveys, qualitative studies, and community engagement within vulnerable communities to fully understand vulnerability. Another key limitation is that the index has not been validated against empirical data. To address this, we plan to validate the index with the VANguard survey results to test how well the index aligns with the observed data and what it predicted. Additionally, we didn’t include data on health facility structural factors such as vaccine stockouts, vaccination staff availability, that would provide additional information on the supply chain and vaccine distribution challenges within districts or counties. Our analysis was limited to correlation and could not establish any casual relationships between the risk factors and vaccine impact. Finally, the equal weight scheme used in the index calculation requires further validation and sensitivity analysis to assess the impact of alternative weighting schemes.

Despite these limitations, the study highlights the multidimensional nature of vulnerability and demonstrates that assessments based on multiple indicators provide a more holistic view than a single indicator or domain. By integrating data on social, structural and biological factors, our approach provides a more holistic assessment of vulnerabilities related to reduced vaccine impact. Thus, the index classification and mapping serve as a starting point for understanding how these factors interact to influence vaccine impact and identify where communities where targeted interventions are most needed. Additionally, the index is adapted to different spatial scales depending on the availability of data, making it’s a versatile tool for identifying vulnerable communities to guide efforts to improve vaccine impact in other settings.

## Conclusion

Maximizing vaccine impact requires a precise understanding of the geographical distribution of vulnerable communities and the underlying factors contributing to their vulnerability. The CVIVI developed in this study offers a starting point for identifying these vulnerable communities and the key challenges they are facing. While this information can guide development of targeted interventions, integrated strategies that address social, structural, and biological factors underlying the vulnerability are required to achieve long term vaccine benefits. By going beyond vaccine coverage, such holistic approaches can ensure that even the most vulnerable fully benefit from vaccination programs and achieve better health outcomes.

## Ethical approval

We sought permission to use the KDHS and UDHS survey data from the MEASURE DHS program website https://www.dhsprogram.com/data/available-datasets.cfm. Ethical approval for the 2022 KDHS was granted by the Institutional Review Board of the Inner-City Fund (ICF). The survey was conducted by the Kenya National Bureau of Statistics in collaboration with other development partners. The protocol for the 2020 Kenya Malaria Indicator Survey (KMIS) was approved by the Kenyatta National Hospital/University of Nairobi Scientific and Ethics Review Committee and the institutional review board at ICF. Further details regarding the conduct of these survey may be found in the 2022 KDHS report ^23^, and 2020 KMIS report ^49^. Ethical approval for the 2016 UDHS was obtained from the Institutional Review Board of ICF and the Uganda National Council for Science and Technology (UNCST). The survey was implemented by the Uganda Bureau of Statistics (UBOS), in collaboration with Ministry of Health. Further details regarding the conduct of the study may be found in the 2016 UDHS report ^22^. For all the surveys, written informed consent was obtained from all human participants and from legally appointed representatives of minor participants. No formal ethical approval was required for this work as it used secondary data from publicly available datasets.

## Data Availability

All the data used in this work is publicly available via the data sources cited in the paper. Data on vulnerability indicators and vaccine coverage in Kenya were obtained from publicly available 2022 KDHS report ^23^, and 2020 KMIS report ^49^. In Uganda, data on social, structural and biological vulnerability indicators was obtained from the UDHS 2016 datasets, accessible on the DHS website https://dhsprogram.com/data/dataset_admin/index.cfm ^70^. To access the data, permission was obtained from the DHS administration through registration and submission of a brief proposal for this study. Access to the datasets was granted within two working days. Immunisation data and malaria prevalence in Uganda were obtained through formal requests from the Uganda Ministry of Health. Shapefiles for Uganda and Kenya used for spatial analysis were freely downloaded from https://gadm.org/maps ^71^ Open Science Framework: Assessing community vulnerability to reduced vaccine impact in Uganda and Kenya: A spatial data analysis. https://doi.org/10.17605/OSF.IO/QBYSJ ^64^ The project contains the following underlying data: Immunisation data Uganda: Excel spreadsheet with data on immunisation performance in Uganda per district for measles, DPT1 and DPT3 vaccines Open Science framework: Assessing community vulnerability to reduced vaccine impact in Uganda and Kenya: A spatial data analysis. https://doi.org/10.17605/OSF.IO/QBYSJ ^64^ The project contains the following extended data: Supplementary material: Additional tables (Table 1: Domain specific and overall vulnerability index scores for counties in Kenya, Table 2: Domain-specific and overall vulnerability index scores for districts in Uganda, Table 3: Summary statistics of vulnerability indicators, Table 4: Estimates of vulnerability indicators across counties in Kenya, Table 5: Estimates of vulnerability indicators across districts in Uganda). Supplementary material: Additional figures (Figure 1: Domain specific vulnerability scores across counties in Kenya, Figure 2: Domain specific vulnerability scores across districts in Uganda, Figure 3: Degree of correlation between the vulnerability index and vaccination coverage at the county level in Kenya, Figure 4: Degree of correlation between the vulnerability index and vaccination coverage at the district level in Uganda). Data are available under the terms of the Creative Commons Zero "No rights reserved" data waiver (CC-BY 4.0)

## Software availability

All statistical analysis, and visualization was carried out in R software (version 4.4.2) available for free download at https://cran.r-project.org/. Geographical preprocessing and spatial maps were generated in Quantum Geographic Information System software (Q-GIS, version 3.38), which is also available for free download at https://www.qgis.org/.

## Code availability

The R code is available at the GitHub link:

https://github.com/Robinah23/Community-Vaccine-Impact-Vulnerability-Index-
