# Supplementary material for: Mapping Community Vulnerability to reduced Vaccine Impact in Uganda and Kenya: A spatial Data-driven Approach
Source: NIHR Open Res. 2025 Sep 23;5:24. Originally published 2025 Mar 17. [Version 3] doi: 10.3310/nihropenres.13898.3 (PMC12572777; doi:10.3310/nihropenres.13898.3)
Supplement: Peer review history

# Reviewer report: Reviewer response for version 3

**DOI:** 10.3310/nihropenres.15354.r37261

**Published:** 2025-10-29

**Authors:** Morufu Olalekan Raimi

**Affiliations:** 1 Deputy Director, Niger Delta Institute for Emerging and Re-Emerging Infectious Diseases (NDIERID), Federal University Otuoke, Otuoke, Bayelsa State, Nigeria

**Mapping Community Vulnerability to reduced Vaccine Impact in Uganda and Kenya: A spatial Data-driven Approach.**

**Overall Recommendation: Accepted**

 This is a well-conceived, rigorously executed, and clearly presented study. The authors have successfully developed a novel and practical tool (the CVIVI) that makes a significant contribution to the field of vaccine equity and public health planning. The manuscript has undergone two rounds of revision, and the authors have been highly responsive to the reviewer's comments, substantially improving the work. The remaining minor issues do not detract from the overall quality, importance, and readiness of the manuscript for Indexing.

**Detailed Review**

**1. Strengths**

- **Novel and Important Concept:** The development of a composite index (CVIVI) that moves beyond mere vaccine *coverage* to assess vulnerability to reduced vaccine *impact* is a significant and timely contribution. It addresses a critical gap by integrating social, structural, *and* biological domains.
- **Robust and Transparent Methodology:** The methodology is clearly described and well-justified. The process, from indicator selection based on literature, data availability, and local relevance, through normalization, percentile ranking, and aggregation is systematic and reproducible.
- **Strong Justification for Analytical Choices:** The authors provides an excellent, evidence-based rationale for their use of equal weighting (citing poor KMO test results for Kenya and lack of outcome data for regression-based weights). This demonstrates thoughtful consideration of alternatives and strengthens the methodological rigor.
- **Excellent Data Visualization and Presentation:** The use of maps, correlation heatmaps, and bar plots effectively communicates complex spatial and multivariate data. The figures are clear, informative, and directly support the results.
- **Action-Oriented and Policy-Relevant:** The discussion and conclusion are not merely descriptive; they effectively translate the findings into concrete policy recommendations and link them to global health agendas like WHO's IA2030. The case studies of Bullisa and Turkana ground the analysis in real-world public health challenges.
- **Comprehensive Revisions:** The authors have thoroughly addressed the previous reviewer's concerns, significantly strengthening the manuscript in its current version (V3). The additions to the methodology, limitations, and policy sections are particularly noteworthy.

**2. Minor Concerns / Suggestions for Polishing**

**a) Data and Limitations:**

- The authors correctly identify the use of older Ugandan data (UDHS 2016) as a limitation. While they have addressed this, it would be beneficial to briefly speculate on the potential direction of bias. For instance, could vulnerability in some districts have worsened or improved since 2016, and how might this affect the current relevance of the Ugandan maps?
- The issue of vaccine coverage >100% in Uganda is noted. The authors' explanation (denominator issues) is standard. A brief sentence acknowledging that this affects the precision of the coverage analysis (but not the CVIVI construction itself) would suffice.

**b) Results and Interpretation:**

- The finding of “no correlation” between CVIVI and vaccine coverage in Uganda is intriguing and well-discussed. To further strengthen this point, the authors could briefly reiterate in the results that this decoupling underscores the core premise of the CVIVI: that high coverage does not automatically equate to high impact, due to the other vulnerability factors.
- In the case study of Kampala (Page 11), it is noted that despite low overall vulnerability, the district has high malaria incidence and limited access to postnatal care. This is a perfect illustration of the value of domain-specific scores. This point could be emphasized more strongly to show how the tool can identify specific, hidden challenges even in “low vulnerability” areas.

**c) Wording and Flow:**

- The manuscript is generally well-written. A final proofread for minor grammatical tweaks and sentence flow is recommended. For example:
  - On Page 4, the phrase “Reduced vaccine impact may result from interplay between structural, social, and biological factors, that limit...” could be slightly rephrased for smoother reading: “Reduced vaccine impact may result from the interplay of structural, social, and biological factors that limit...”
  - On Page 17, the sentence “This highlights that relying solely on vaccine coverage estimates...” is cut off and repeated. This is a minor formatting error that should be corrected.

**3. Points Clarified by the Revisions**

 The authors' responses in the revision history have fully addressed what were previously major concerns:

- **Indicator Selection:** The rationale is now comprehensive, covering literature, local relevance, prevalence, variability, and data availability.
- **Weighting:** The explanation for choosing equal weights over PCA or regression-based methods is now thorough and convincing.
- **Spatial Data Handling:** The process of harmonizing names and handling new districts is clearly described.
- **Policy Linkage:** The conclusions are now explicitly and effectively linked to the WHO IA2030 framework.

**Decision Justification**

 This manuscript is **recommended for acceptance**. It represents a high-quality piece of research that is methodologically sound, novel in its approach, and of immediate practical utility for public health policymakers and planners in Uganda, Kenya, and potentially other similar contexts. The authors have demonstrated a commendable ability to incorporate critical feedback, resulting in a manuscript that is clear, robust, and impactful. The minor suggestions listed above are for further polishing and do not warrant another revision cycle.

**Final Comments**

 This study provides a powerful, data-driven framework for moving beyond blanket immunization strategies and towards targeted, context-specific interventions. The Community Vaccine Impact Vulnerability Index (CVIVI) is a valuable tool for ensuring that the goal of "leaving no one behind" in vaccination programs is met with precision and insight. The authors are to be congratulated on a fine piece of work.

 Recommendation: Accepted

**Sincerely,Fpubli**

**Dr. Morufu Olalekan RAIMI,**

Is the work clearly and accurately presented and does it cite the current literature?

Yes

If applicable, is the statistical analysis and its interpretation appropriate?

Partly

Are all the source data underlying the results available to ensure full reproducibility?

Yes

Is the study design appropriate and is the work technically sound?

Partly

Are the conclusions drawn adequately supported by the results?

Yes

Are sufficient details of methods and analysis provided to allow replication by others?

Partly

Reviewer Expertise:

Environmental Epidemiology, Health Economics, Pollution Control Management, Hydrogeochemistry, Groundwater Pollution, Groundwater Quality, Health Risk Assessment, Water Pollution and Management, Water and Gender, Chemicals & Health, Environmental Public Health Policy & Practice, Environmental Justice & Health Equity, Noise & Health, Carbon Accounting Services, Environmental & Health Impact Assessments, Community based participatory Research, Climate Change Mitigation and Adaptation, Oil Spills Clean up and Remediation, Environmental Toxicology & Health, Emissions Control and Respiratory Protection, Biogeochemistry, Earth sciences - Atmospheric sciences, Earth sciences - Climate change, Earth sciences – Limnology, Health care - Health education, Health care - Health policy, Health care - Health services research, Pollution research and control, Public health and epidemiology - Biostatistics and methodsPublic health and epidemiology - Chronic diseases, Public health and epidemiology - Environmental health, Public health and epidemiology – General, Public health and epidemiology - Global health, Public health and epidemiology - Health behavior, health promotion and society, Public health and epidemiology - Health policies, systems and management, Public health and epidemiology - Occupational health

I confirm that I have read this submission and believe that I have an appropriate level of expertise to confirm that it is of an acceptable scientific standard.

# Reviewer report: Reviewer response for version 3

**DOI:** 10.3310/nihropenres.15354.r37809

**Published:** 2025-10-16

**Authors:** Rita Barradas Barata

**Affiliations:** 1 Faculdade de Ciências Médicas da Santa Casa de São Paulo, Sao Paulo, Brazil

I consider the article well-written, with clear objectives, a methodology appropriate for achieving the proposed goals, the use of secondary data from national surveys, and a simple and objective analysis that is easily applicable to health professionals and has sufficient discriminatory capacity to guide public health interventions. The presentation of the results and the discussion are complete and accurate, detailing all the important aspects for understanding the findings.

 I value the detailed description of the methodological procedures and the choice of relatively simple approaches for constructing the vulnerability indicator. Although based on the assignment of positions, it achieves its intended purpose without requiring more sophisticated computer and statistical resources. Considering the use of this model by health professionals without academic training and education, I believe its simplicity is an invaluable advantage.

 I recommend the article to people primarily interested in applying public health principles to solve population-based problems. The experience of low- and middle-income countries can also be very useful in addressing marginalized and impoverished communities, even in high-middle income countries or high income countries.

Is the work clearly and accurately presented and does it cite the current literature?

Yes

If applicable, is the statistical analysis and its interpretation appropriate?

Yes

Are all the source data underlying the results available to ensure full reproducibility?

Yes

Is the study design appropriate and is the work technically sound?

Yes

Are the conclusions drawn adequately supported by the results?

Yes

Are sufficient details of methods and analysis provided to allow replication by others?

Yes

Reviewer Expertise:

Infectious diseases epidemiology; vaccines; social health inequalities; scientific police

I confirm that I have read this submission and believe that I have an appropriate level of expertise to confirm that it is of an acceptable scientific standard.

# Reviewer report: Reviewer response for version 2

**DOI:** 10.3310/nihropenres.15272.r36384

**Published:** 2025-08-20

**Authors:** Morufu Olalekan Raimi

**Affiliations:** 1 Deputy Director, Niger Delta Institute for Emerging and Re-Emerging Infectious Diseases (NDIERID), Federal University Otuoke, Otuoke, Bayelsa State, Nigeria

**Title**: Mapping Community Vulnerability to reduced Vaccine Impact in Uganda and Kenya: A spatial Data-driven Approach

**Journal**: NIHR Open Research

**Recommendation**: Accepted with Minor Revisions

**Reviewer**: Prof. RAIMI Morufu Olalekan

**Review Date: 15/08/2025**

**Reviewer Comments:**

 Dear Authors,

 Thank you for submitting your manuscript titled *“Mapping Community Vulnerability to Reduced Vaccine Impact in Uganda and Kenya: A Spatial Data-driven Approach.”* This study provides a significant contribution to understanding the multidimensional vulnerabilities that influence vaccine effectiveness in two African countries, Uganda and Kenya. By developing the Community Vaccine Impact Vulnerability Index (CVIVI), the study presents a novel approach to identifying vulnerable communities and can guide targeted interventions to improve vaccination outcomes. However, few areas need further clarification and refinement to meet the publication standards.

**i. Title**

- **Current Title:** *Mapping Community Vulnerability to Reduced Vaccine Impact in Uganda and Kenya: A Spatial Data-driven Approach*. The title maintains the study's focus, making it more concise and directly reflective of the methodology.

**ii. Abstract**

- The abstract is well structured to enhance clarity. It currently provides useful information.

**iii. Keywords**

- The keywords are relevant

**iv. Introduction**

- The introduction provides a solid background,

**v. Novelty and Contribution**

- The manuscript contributes significantly by integrating social, structural, and biological factors into a vulnerability index for vaccine impact.

**vi. Methodology**

- The methodology is robust, but more details are needed to understand the data sources and the rationale behind indicator selection and weighting. Author should explain in more detail why certain indicators were chosen and how their relevance was determined beyond the literature review; Provide justification for the use of equal weighting in the index construction, and whether alternative weighting methods were considered; Clarify the rationale for using secondary data and address any limitations regarding data consistency, particularly with vaccine coverage estimates over 100% as well as Discuss the spatial data handling process more thoroughly, particularly regarding potential geographic biases.

**vii. Results and Data Presentation**

- The results are well-organized.

**viii. Discussion and Interpretation**

- The discussion offers valuable insights

**ix. Conclusion and Recommendations**

- The conclusion summarizes the study well but could be more action-oriented with specific policy recommendations by linking these recommendations more explicitly to broader public health goals, such as those outlined in the WHO Immunization Agenda 2030.

**x. References**

- The reference list is generally well-developed,

**Conclusion**

 This is a highly promising study with significant potential for improving our understanding of vaccine impact in vulnerable communities. With a few revisions to clarify the methodology, and provide more actionable recommendations, it could make a substantial contribution to public health research and policy. I look forward to seeing the revised manuscript.

**Recommendation**: **Accepted with Minor Revisions**

Is the work clearly and accurately presented and does it cite the current literature?

Yes

If applicable, is the statistical analysis and its interpretation appropriate?

Partly

Are all the source data underlying the results available to ensure full reproducibility?

Yes

Is the study design appropriate and is the work technically sound?

Partly

Are the conclusions drawn adequately supported by the results?

Yes

Are sufficient details of methods and analysis provided to allow replication by others?

Partly

Reviewer Expertise:

Environmental Epidemiology, Health Economics, Pollution Control Management, Hydrogeochemistry, Groundwater Pollution, Groundwater Quality, Health Risk Assessment, Water Pollution and Management, Water and Gender, Chemicals & Health, Environmental Public Health Policy & Practice, Environmental Justice & Health Equity, Noise & Health, Carbon Accounting Services, Environmental & Health Impact Assessments, Community based participatory Research, Climate Change Mitigation and Adaptation, Oil Spills Clean up and Remediation, Environmental Toxicology & Health, Emissions Control and Respiratory Protection, Biogeochemistry, Earth sciences - Atmospheric sciences, Earth sciences - Climate change, Earth sciences – Limnology, Health care - Health education, Health care - Health policy, Health care - Health services research, Pollution research and control, Public health and epidemiology - Biostatistics and methodsPublic health and epidemiology - Chronic diseases, Public health and epidemiology - Environmental health, Public health and epidemiology – General, Public health and epidemiology - Global health, Public health and epidemiology - Health behavior, health promotion and society, Public health and epidemiology - Health policies, systems and management, Public health and epidemiology - Occupational health

I confirm that I have read this submission and believe that I have an appropriate level of expertise to confirm that it is of an acceptable scientific standard, however I have significant reservations, as outlined above.

# Response

**Published:** 2025-09-18

**Authors:** Robinah Nalwanga

**Affiliations:** Immunomodulation and Vaccines, Medical Research Council/Uganda Virus Research Institute and London School of Hygiene & Tropical Medicine Uganda Research Unit, Kampala Entebbe, Uganda

**Manuscript Title:** Mapping Community Vulnerability to Reduced Vaccine Impact in Uganda and Kenya: A Spatial Data-driven Approach

 Dear Reviewer,

 We would like to sincerely thank you for your thoughtful and constructive feedback provided on our manuscript. We are encouraged by the positive comments regarding the novelty, methodological rigor, and contribution of our work. We have carefully revised the manuscript in response to the reviewer’s suggestions and provide detailed responses below. All changes have been incorporated into the revised version, with page numbers indicated where appropriate.

**Reviewer Comments and Author Responses**

**i. Title**

 Current Title: *Mapping Community Vulnerability to Reduced Vaccine Impact in Uganda and Kenya: A Spatial Data-driven Approach*. The title maintains the study's focus, making it more concise and directly reflective of the methodology.

*We thank the reviewer for this positive feedback. No change required*

**ii. Abstract**

 The abstract is well structured to enhance clarity. It currently provides useful information.

*We thank the reviewer for this positive comment.*

**iii. Keywords**

 The keywords are relevant

*We thank the reviewer for this positive comment.*

**iv. Introduction**

 The introduction provides a solid background

*We thank the reviewer for this positive comment.*

**v. Novelty and Contribution**

 The manuscript contributes significantly by integrating social, structural, and biological factors into a vulnerability index for vaccine impact.

*We thank the reviewer for recognizing the novelty and contribution of our study.*

**vi. Methodology**

 The methodology is robust, but more details are needed to understand the data sources and the rationale behind indicator selection and weighting. Author should explain in more detail why certain indicators were chosen and how their relevance was determined beyond the literature review.

*We thank the reviewer for this comment. We have now expanded the methodology to clarify our rationale for indicator selection. In the addition to literature evidence, we considered the local relevance of each indicator and its prevalence and variability across communities to ensure it could meaningfully distinguish levels of vulnerability. Given the limited availability of quality data at district/county level, we prioritised indicators for which reliable data existed. (see Indicator selection subsection, page 6).*

 Provide justification for the use of equal weighting in the index construction, and whether alternative weighting methods were considered

*We appreciate the reviewer for this suggestion. We considered alternative weighting approaches such as PCA and regression-based methods. However, PCA was unsuitable because the data for Kenya had poor adequacy (KMO = 0.50), making results unreliable and not comparable across countries. Regression-based weights were also not feasible due to limited and inconsistent outcome data on vaccine coverage and lack of data on immune response. To avoid introducing bias, we therefore retained equal weighting, which we believe provides the most robust and transparent approach. The methods section has been revised to reflect these considerations (see weighting subsection, page 11-12).*

 Clarify the rationale for using secondary data and address any limitations regarding data consistency, particularly with vaccine coverage estimates over 100%

*We thank the reviewer for this comment.  We used secondary data (mainly DHS survey data) because they provide comprehensive nationwide population level information that would be difficult and costly to collect prospectively across multiple districts or counties in Uganda and Kenya. Other limitations of the DHS data have been added to the manuscript. For vaccine coverage particularly for Uganda, we used administrative records obtained from the Ministry of Health. Some estimates exceeded 100%, likely due to inaccurate population denominators. We requested updated 2022 Census data to recalculate coverage, though we were not successful as this data are not yet publicly available. Importantly, vaccine coverage data were not used in calculating the vulnerability index but rather to validate it, examining whether areas identified as vulnerable had lower coverage. Other limitations of secondary data are described in the manuscript.*

 Discuss the spatial data handling process more thoroughly, particularly regarding potential geographic biases.

*We appreciate the reviewer for this suggestion. We harmonised district and county names across the indicator datasets and shapefiles. To address boundary changes emerging from the newly created districts, we excluded data for the districts that were missing.*

**vii. Results and Data Presentation**

 The results are well-organized.

*We thank the reviewer for this positive feedback.*

**viii. Discussion and Interpretation**

 The discussion offers valuable insights

*We thank the reviewer for this positive comment.*

**ix. Conclusion and Recommendations**

 The conclusion summarizes the study well but could be more action-oriented with specific policy recommendations by linking these recommendations more explicitly to broader public health goals, such as those outlined in the WHO Immunization Agenda 2030.

*We appreciate this comment. We have revised the conclusion as per your suggestion.*

**x. References**

 The reference list is generally well-developed

*We thank the reviewer for this positive comment*

 .

# Reviewer report: Reviewer response for version 1

**DOI:** 10.3310/nihropenres.15105.r35021

**Published:** 2025-03-25

**Authors:** Morufu Olalekan Raimi

**Affiliations:** 1 Deputy Director, Niger Delta Institute for Emerging and Re-Emerging Infectious Diseases (NDIERID), Federal University Otuoke, Otuoke, Bayelsa State, Nigeria

Review of Manuscript ID: 13898 (Robinah Nalwanga et al.)

 Journal: NIHR Open Research

 Recommendation: Accepted with Major Revisions

 My Overall Evaluation

 The manuscript, *“Assessing Community Vulnerability to Reduced Vaccine Impact in Uganda and Kenya: A Spatial Data Analysis,”* presents an important contribution to public health by introducing the Community Vaccine Impact Vulnerability Index (CVIVI). The study effectively integrates structural, social, and biological factors to identify regions at risk of reduced vaccine effectiveness. However, several areas require substantial revisions to enhance clarity, methodological rigor, and policy relevance.

 Key Review Points:

 1. Title Refinement:

- The title is descriptive but could better emphasize the spatial analysis aspect.
- Suggested revision: *“Mapping Community Vulnerability to Reduced Vaccine Impact in Uganda and Kenya: A Spatial Data-Driven Approach.”*

2. Abstract Improvements:

- The abstract is well-structured but lacks key specifics. Author should specify the number of districts/counties analyzed, Indicate the primary data sources used for constructing the CVIVI and provide a stronger conclusion by linking findings to policy recommendations.

3. Keywords:

- The keywords should be expanded to include *“Spatial Epidemiology,”* *“Vaccine Equity,”* and *“Public Health Vulnerability Mapping.”*

4. Introduction:

- Author should provide strong background on vaccine disparities in Africa and more discussion on previous vulnerability indices used in public health is needed. Clearly define how this study builds on or differs from existing vaccine impact models.

5. Novelty and Contribution:

- The manuscript introduces a novel approach with the CVIVI, but the discussion of its practical application is limited. Authors should clarify how policymakers or healthcare practitioners can use the CVIVI for decision-making.

6. Methodology:

- The methodology is well-detailed but lacks clarity on certain aspects.
  - How were the vulnerability indicators weighted?
  - What geospatial techniques were used for vulnerability classification?
  - How was correlation analysis conducted between indicators?
- A justification for using percentile ranking over other methods (e.g., principal component analysis) should be provided.

7. Results:

- The results are interesting but require additional details. Author should provide summary statistics for each vulnerability domain. Author should use more tables or visualizations to represent vulnerability levels as well as Include case studies of highly vulnerable districts to illustrate the real-world impact.

8. Discussion:

- The discussion lacks a detailed comparison with similar vulnerability indices used in other countries.
- Policy implications should be explored in greater depth, particularly in relation to vaccination program targeting.
- The potential limitations of using secondary data sources should be addressed.

9. Conclusion and Recommendations:

- The conclusion is informative but could be more impactful. Author should summarize how the findings can guide targeted interventions, provide explicit recommendations for policymakers, such as strengthening outreach programs in identified high-risk regions.

10. References:

- The reference list should include more studies on geospatial health analysis.
- Recent literature on vaccine equity and social determinants of health should be incorporated.

Dr. Morufu Olalekan RAIMI,

 BSc, *(Geography. and Environmental Management), *Diploma. *(Environmental Health), *M.Sc. *Environmental Health Management), *M.Phil. *(Environmental Health Science), *P.hD *(Environmental Health Science), *MNES, REHO, LEHO, FAIWMES

Is the work clearly and accurately presented and does it cite the current literature?

Yes

If applicable, is the statistical analysis and its interpretation appropriate?

Partly

Are all the source data underlying the results available to ensure full reproducibility?

Yes

Is the study design appropriate and is the work technically sound?

Partly

Are the conclusions drawn adequately supported by the results?

Yes

Are sufficient details of methods and analysis provided to allow replication by others?

Partly

Reviewer Expertise:

Environmental Epidemiology, Health Economics, Pollution Control Management, Hydrogeochemistry, Groundwater Pollution, Groundwater Quality, Health Risk Assessment, Water Pollution and Management, Water and Gender, Chemicals & Health, Environmental Public Health Policy & Practice, Environmental Justice & Health Equity, Noise & Health, Carbon Accounting Services, Environmental & Health Impact Assessments, Community based participatory Research, Climate Change Mitigation and Adaptation, Oil Spills Clean up and Remediation, Environmental Toxicology & Health, Emissions Control and Respiratory Protection, Biogeochemistry, Earth sciences - Atmospheric sciences, Earth sciences - Climate change, Earth sciences – Limnology, Health care - Health education, Health care - Health policy, Health care - Health services research, Pollution research and control, Public health and epidemiology - Biostatistics and methodsPublic health and epidemiology - Chronic diseases, Public health and epidemiology - Environmental health, Public health and epidemiology – General, Public health and epidemiology - Global health, Public health and epidemiology - Health behavior, health promotion and society, Public health and epidemiology - Health policies, systems and management, Public health and epidemiology - Occupational health

I confirm that I have read this submission and believe that I have an appropriate level of expertise to confirm that it is of an acceptable scientific standard, however I have significant reservations, as outlined above.

# Response

**Published:** 2025-07-14

**Authors:** Robinah Nalwanga

**Affiliations:** Immunomodulation and Vaccines, Medical Research Council/Uganda Virus Research Institute and London School of Hygiene & Tropical Medicine Uganda Research Unit, Kampala Entebbe, Uganda

**Key Review Points:**

 1. Title Refinement: The title is descriptive but could better emphasize the spatial analysis aspect. Suggested revision: “Mapping Community Vulnerability to Reduced Vaccine Impact in Uganda and Kenya: A Spatial Data-Driven Approach.”

*Thanks for this suggestion, we have changed the title of the manuscript as suggested.*

 2. Abstract Improvements: The abstract is well-structured but lacks key specifics. Author should specify the number of districts/counties analyzed, Indicate the primary data sources used for constructing the CVIVI and provide a stronger conclusion by linking findings to policy recommendations.

*Thanks for your comment. We have now included additional information as requested in the abstract.*

 3. Keywords: The keywords should be expanded to include “Spatial Epidemiology,” “Vaccine Equity,” and “Public Health Vulnerability Mapping.”

*Thanks for addition of key words. I have added more key words.*

 4. Introduction: Author should provide strong background on vaccine disparities in Africa and more discussion on previous vulnerability indices used in public health is needed. Clearly define how this study builds on or differs from existing vaccine impact models.

*Thank you for your comment, we have revised the introduction as per your comments and suggestions*.

 5. Novelty and Contribution: The manuscript introduces a novel approach with the CVIVI, but the discussion of its practical application is limited. Authors should clarify how policymakers or healthcare practitioners can use the CVIVI for decision-making.

*Thanks for this suggestion, we included subsection on the practical application of the CVIVI and clarified how the index can support policy, markers with decision making*

 6. Methodology: The methodology is well-detailed but lacks clarity on certain aspects.

 How were the vulnerability indicators weighted?

*We applied equal weight for the vulnerability indicators. Firstly, a percentile rank was calculated for each vulnerability indicator. Then we compute a domain specific vulnerability score by averaging the percentile rank for each indicator in each domain (this was done by summing the percentile ranks for each domain and dividing by the number of individuals in the respective domain). Then equal weights were applied to compute the overall vulnerability index, computed by averaging the three domain specific scores. This is described in the Weighting section of the Methods (refer to line 307).*

 What geospatial techniques were used for vulnerability classification?

*We used the quantile classification method in QGIS, where the districts/counties were grouped into five quartiles based on their CVIVI score.*

 How was correlation analysis conducted between indicators?

*We computed pairwise correlations between each pair of indicators, using Pearson’s correlation coefficient as data were normally distributed. This is described in the Statistical Analysis section of the Method (refer to statistical analysis section under results- line 348).*

 A justification for using percentile ranking over other methods (e.g., principal component analysis) should be provided.

*Percentile ranking was first used to scale each individual indicator, so that they were all on the same scale (0-1). We did consider using principal components analysis to determine which weights should then be applied to each scaled indicator, when combining them into a single metric, but instead chose to use an equal weighting approach which is agnostic to the relative importance of different indicators*.

 7. Results:

 The results are interesting but require additional details. Author should provide summary statistics for each vulnerability domain.

*Thanks for this suggestion. We have presented a table on summary statistics for each   vulnerability domain.*

 Author should use more tables or visualizations to represent vulnerability levels as well as Include case studies of highly vulnerable districts to illustrate the real-world impact.

*Thanks for your comment, this very important. However, we presented spatial maps showing different vulnerability levels for the overall vulnerability index in Uganda (refer to Figure 4) and Kenya (refer to Figure 5) in the results section. We request for some clarity on the use of more tables.*

 8. Discussion:

 The discussion lacks a detailed comparison with similar vulnerability indices used in other countries. *Thanks for your comment. We have revised the discussion to include comparison of the CVIVI with other indices. Refer to line 555 and line 565 in the discussion section.*

 Policy implications should be explored in greater depth, particularly in relation to vaccination program targeting. *Thanks for your comment. We have revised and included a separate subsection on policy implications (refer to line 633).*

 The potential limitations of using secondary data sources should be addressed.

*Thanks for your comment: We have revised the limitation of secondary data sources as suggested*

 9. Conclusion and Recommendations: The conclusion is informative but could be more impactful. Author should summarize how the findings can guide targeted interventions, provide explicit recommendations for policymakers, such as strengthening outreach programs in identified high-risk regions.

*Thank you for this comment, we have revised the conclusion section to incorporate your suggestions.*

 10. References: The reference list should include more studies on geospatial health analysis. Recent literature on vaccine equity and social determinants of health should be incorporated.

*Thanks for your comment, we have included more references on geospatial health analysis in the discussion and introduction.  We have included more recent literature review on vaccine equity and social determinants of health.*
